# Targeting FLT3 in Acute Myeloid Leukemia: Structural Insights and Key Challenges

**DOI:** 10.1021/acsbiomedchemau.6c00021

**Published:** 2026-04-20

**Authors:** Michele Rodrigues da Silva, Lídia Moreira Lima, Maria Letícia de Castro Barbosa

**Affiliations:** 1 Laboratory of Evaluation and Synthesis of Bioactive Substances (LASSBio), Institute of Biomedical Sciences, 28125 Federal University of Rio de Janeiro, Rio de Janeiro, Rio de Janeiro 21941-902, Brazil; ‡ Graduate Program in Pharmacology and Medicinal Chemistry, Institute of Biomedical Sciences, Federal University of Rio de Janeiro, Rio de Janeiro, Rio de Janeiro 21941-902, Brazil; § Department of Pharmaceutical Sciences, Faculty of Pharmacy, Federal University of Juiz de Fora, Juiz de Fora, Minas Gerais 36036-900, Brazil

**Keywords:** FMS-like tyrosine kinase 3, FLT3 inhibitors, Acute Myeloid Leukemia, AML, FLT3 mutation, FLT3 resistance, drug development, protein kinase, drug resistance, receptor tyrosine kinase

## Abstract

Mutations in FLT3 (FMS-like tyrosine kinase 3) are directly related to the development of acute myeloid leukemia (AML), contributing to the dysregulated proliferation and survival of malignant myeloid cells. FLT3 inhibitors (FLT3i), such as gilteritinib and quizartinib, have demonstrated relevant clinical benefits, including symptom relief and increased overall survival in patients with AML. However, the duration of response to FLT3i remains limited due to the emergence of resistance. In this scenario, the development of targeted therapies has emerged as a promising strategy to overcome these limitations, aiming to address the restrictions observed in the previous generation. These approaches range from the combination of FLT3i with other antileukemic agents to the use of multitarget inhibitors capable of simultaneously modulating FLT3 and other relevant molecular targets, as well as the development of next-generation inhibitors that are more selective and capable of maintaining activity against secondary mutations, such as the gatekeeper mutation (F691L), which confers resistance to clinically available drugs. This review article addresses the therapeutic relevance of FLT3i in AML and provides an overview of the binding modes of representative FLT3 inhibitors. Structural and conformational aspects involved in the interaction with this target, with emphasis on the ATP-binding site, as well as the impact of resistance-associated mutations, are discussed. In addition, this work seeks to correlate the binding modes described in the literature with experimental activity data.

## Graphical abstract

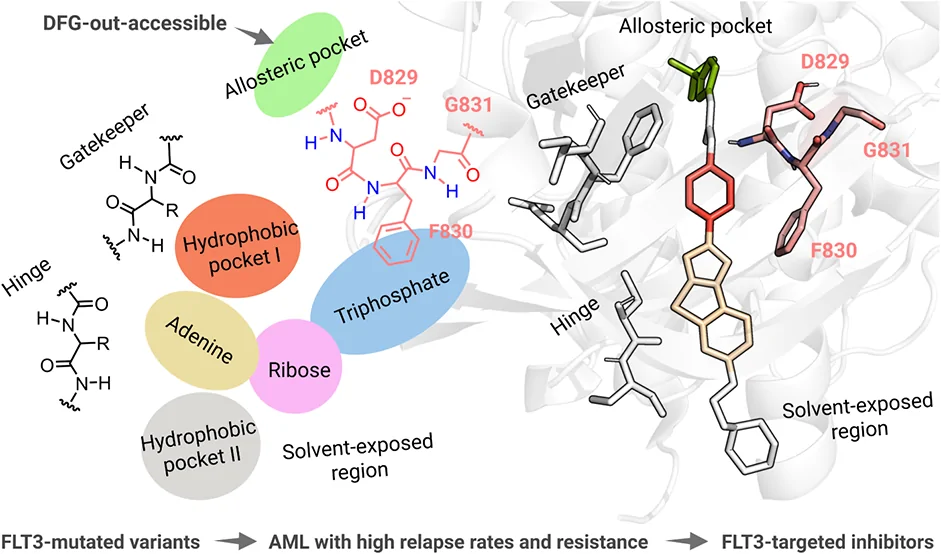

## Introduction

The primary function of protein kinases is to catalyze the transfer of a phosphate group to a substrate by transferring the γ-phosphate group from adenosine triphosphate (ATP). This process, known as phosphorylation, acts as a signal transduction mechanism within the cell and triggers a wide range of responses that regulate diverse cellular activities. Kinases are called serine-threonine kinases when the phosphate group is transferred to serine or threonine residues, and tyrosine kinases when phosphorylation occurs on tyrosine residues. They can also be classified by their organization and cellular localization, being divided into receptor kinases, transmembrane enzymes that require binding of an extracellular ligand for activation, and nonreceptor kinases, which are located in the cytoplasm.^1–4^

Within this context, FMS-like tyrosine kinase 3 (FLT3), a receptor tyrosine kinase (RTK), can be highlighted as a validated therapeutic target for the treatment of acute myeloid leukemia (AML). FLT3 was first identified and isolated by Rosnet et al. in 1991.^5^ This RTK is composed of an extracellular ligand-interaction portion containing five immunoglobulin-like domains, a transmembrane domain, a juxtamembrane domain, and a highly conserved intracellular kinase domain. The kinase domain of the FLT3 receptor is the region responsible for its catalytic activity, which is characterized by two lobes or subdomains. The N-terminal lobe comprises a series of β-strands and an α-helix, known as the C-helix, while the larger C-terminal lobe is predominantly α-helical. The two lobes are connected by the ATP-binding region, referred to as the *hinge*, a highly flexible segment. In addition to these structures, the kinase domain also contains a fragment called the activation loop (A-loop). The A-loop plays a fundamental role in regulating kinase activity, determining whether the enzyme adopts the active (DFG-in) or inactive (DFG-out) conformation (Figure 1).^1,6–9^

**Figure 1:**
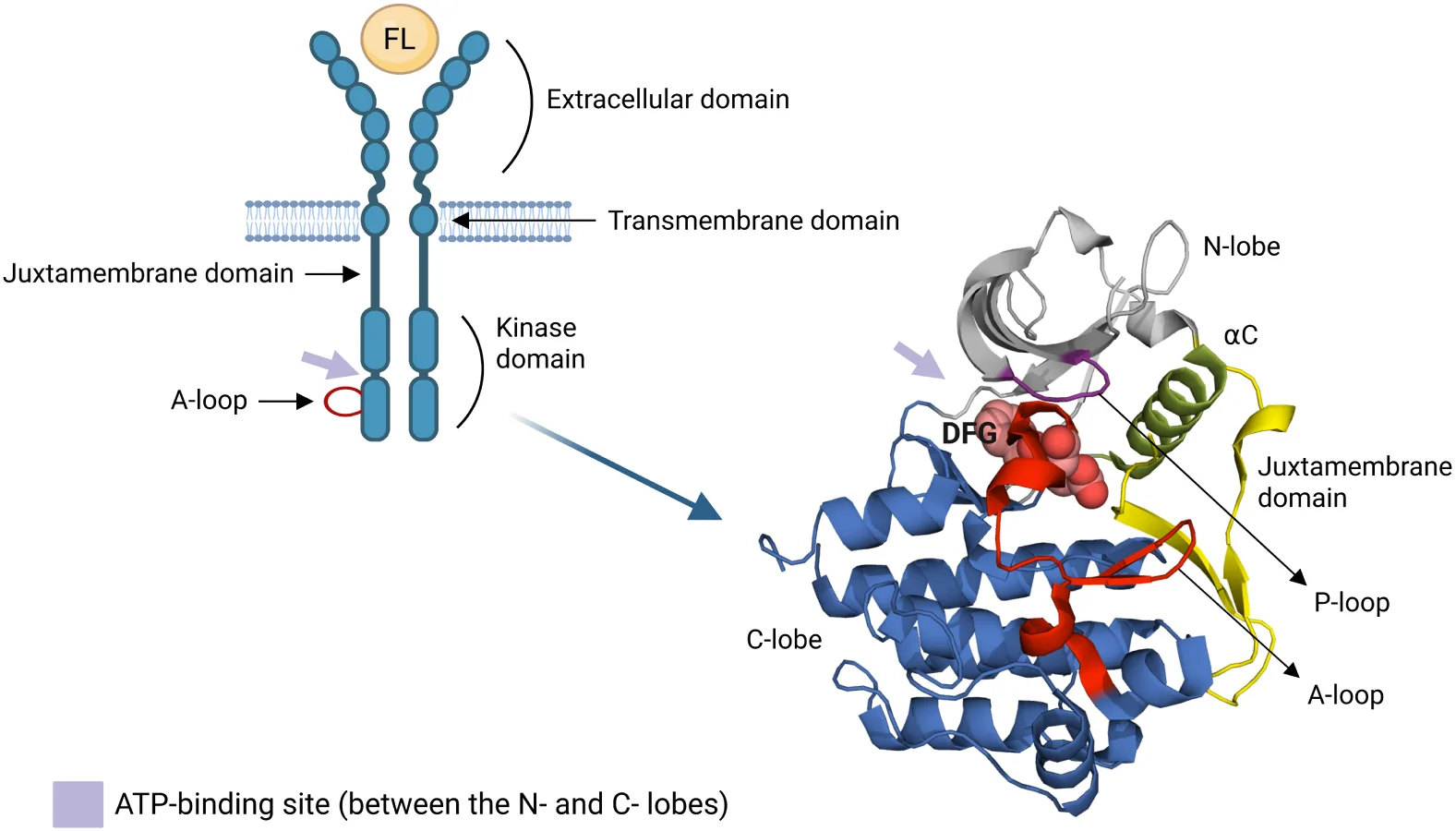
Structure of the FLT3 receptor tyrosine kinase (PDB: 1RJB). Created with BioRender (https://biorender.com/1q66lid).

FLT3 is expressed in various lympho-hematopoietic cells and tissues, playing an essential role in the normal development of stem cells and the immune system. Activation of FLT3 occurs through a process called dimerization, which is initiated when the FLT3 ligand (FL) binds to the receptor’s extracellular domain.^10,11^ The resulting activation triggers downstream signaling pathways such as signal transducer and activator of transcription 5A (STAT5A), the Ras/mitogen-activated protein kinase (MAPK) pathway, and phosphatidylinositol-3-kinase (PI3K)/Akt. These pathways promote the growth, proliferation, survival, and differentiation of myeloid cells.^12–15^

AML is a malignant clonal disorder characterized by the uncontrolled proliferation of myeloid blasts in the bone marrow. It is the most common form of acute leukemia in adults and the deadliest, accounting for approximately 80% of cases, with a median age at diagnosis of 68 years. Alterations in the FLT3 gene, which encodes the receptor tyrosine kinase FLT3, are directly associated with the development of AML, contributing to the dysregulated proliferation and survival of malignant myeloid cells. FLT3 is present in the majority of tumor cells, and FLT3 mutations are found in approximately 30% of AML cases. These mutations influence disease behavior and serve as therapeutic targets, making FLT3 alterations common and clinically relevant in acute myeloid leukemia.^16–18^

## FLT3 Gene Mutations

Mutations in FLT3 can lead to its constitutive activation, independent of external signaling. Among these mutations, two main types stand out, internal tandem duplications (ITD) in the juxtamembrane domain, detected in approximately 25% of patients, and point mutations in residues of the tyrosine kinase domain (TKD), observed in about 5% of cases, particularly at residue D835 (e.g., D835Y, D835V, D835H, D835E, and D835N). The oncogenic FLT3-ITD variant is the most prevalent in the clinical setting and is associated with poorer prognosis. In this mutation, a segment of DNA within the FLT3 gene is duplicated, causing a structural alteration that renders the receptor constitutively active and capable of self-activation in the absence of an external signal such as ligand binding.^7,9,19–23^

FLT3-ITD mutations are typically primary, pretreatment alterations that drive leukemia development and are clinically associated with high relapse rates and reduced overall survival, with their prognostic impact influenced by mutant allele burden and co-occurring mutations. The activation of FLT3 downstream signaling pathways, such as PI3K/Akt, upregulates antiapoptotic proteins like MCL-1 (myeloid cell leukemia-1) and BCL-2 (B-cell lymphoma protein 2), thereby increasing leukemic cell resistance to apoptosis. Dysregulated activation of these pathways contributes to uncontrolled proliferation, increased aggressiveness, and accumulation of leukemic blasts in the bone marrow and peripheral blood, hallmarks of AML (Figure 2).^24–26^

**Figure 2:**
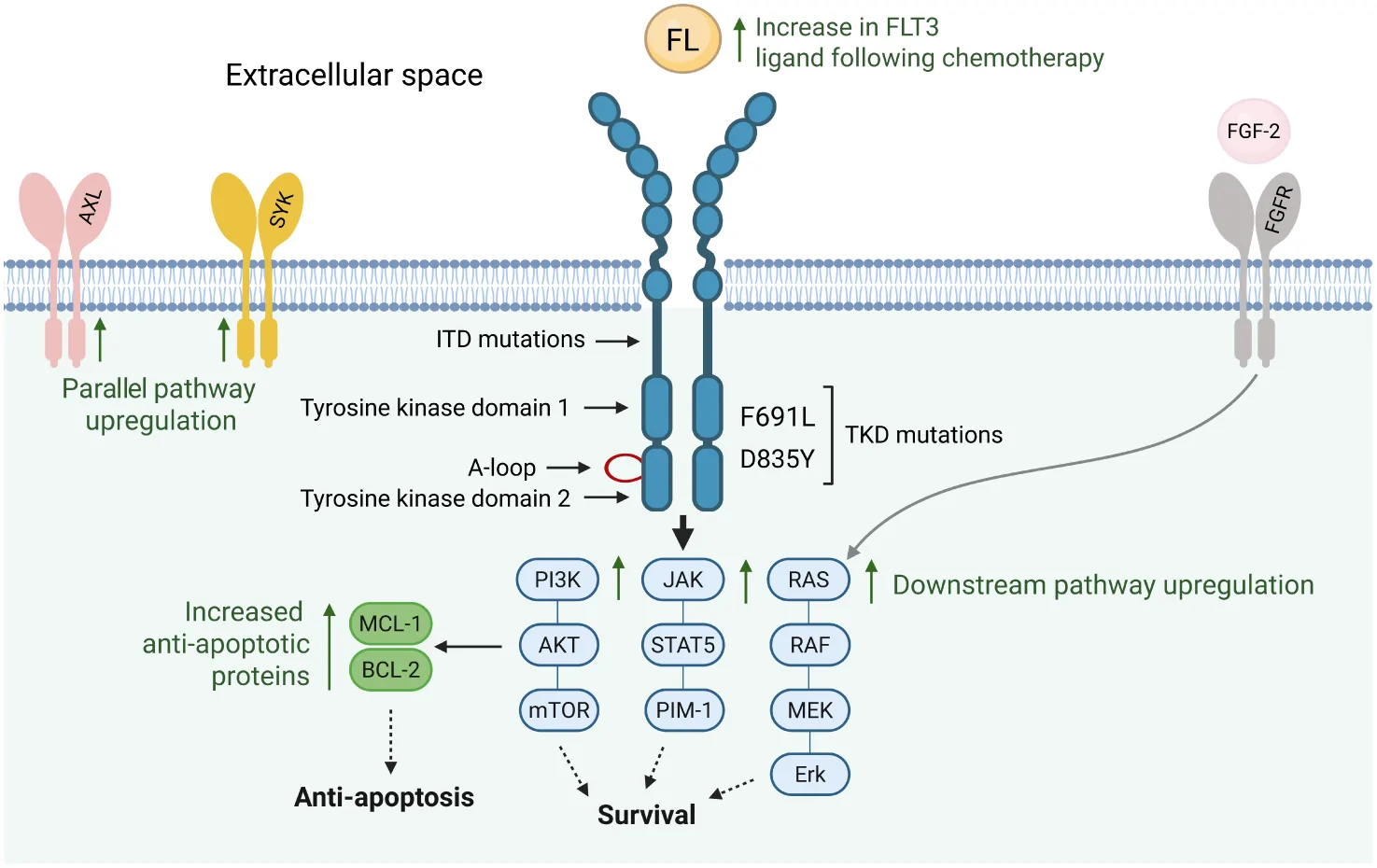
Signaling Pathways and Clinically Relevant Mutations in FLT3. Created with BioRender (https://biorender.com/tz9h95u).

Similar to ITD mutations, FLT3-TKD mutations can induce ligand-independent receptor activation by stabilizing the active conformation of the kinase domain, leading to constitutive activation and triggering FLT3-associated signaling pathways. These are secondary mutations that typically arise after treatment with FLT3 inhibitors (FLT3i), contributing to the development of therapeutic resistance.^6,7,24,26^

## FLT3 Inhibitors

The significance of FLT3 and its mutations in acute myeloid leukemia has established FLT3 inhibitors (FLT3i) as a promising therapeutic strategy. These inhibitors target the ATP-binding site within the intracellular tyrosine kinase domain. Several FLT3i are currently used clinically for AML treatment and are generally classified as first- or second-generation inhibitors.^6,24,27–30^ First-generation inhibitors are nonselective kinase inhibitors, such as midostaurin (PKC412) (**1**), the first FLT3i approved by the U.S. Food and Drug Administration (FDA) for the treatment of AML, acting as an inhibitor of FLT3 and other related kinases.^31,32^ Although they have proven useful in AML treatment, first-generation drugs are commonly associated with adverse effects due to their low selectivity. In contrast, second-generation drugs are more selective for FLT3 and its clinically relevant mutants, targeting fewer off-target proteins and thus exhibiting improved patient tolerability. Examples include gilteritinib (ASP2215) (**2**) and quizartinib (AC220) (**3**). Both generations of FLT3i exhibit significant inhibitory effects on the FLT3-ITD mutation (Figure 3).^26,33–36^

**Figure 3:**
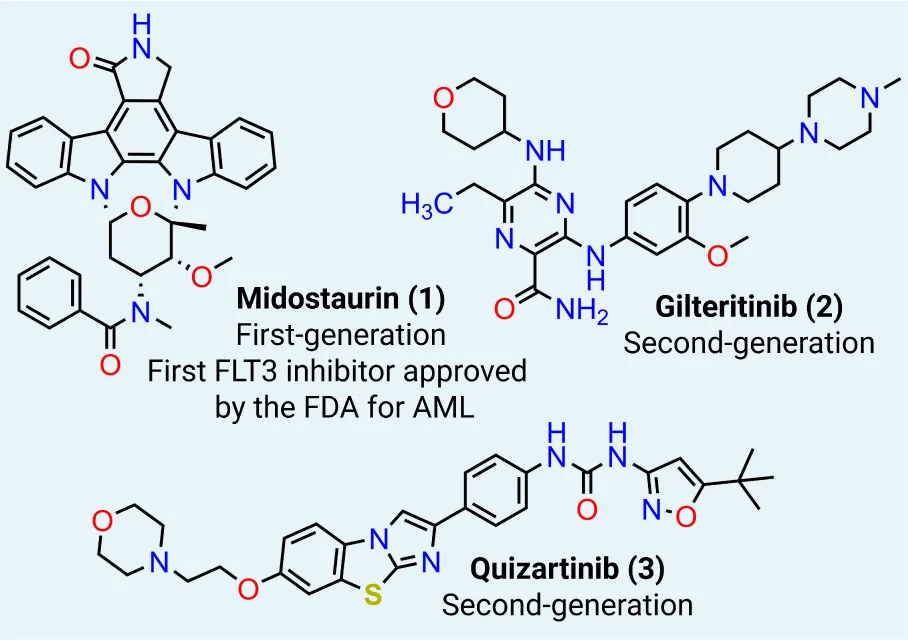
FLT3 inhibitors approved for AML treatment.

Other FLT3i have demonstrated antileukemic effects and are being investigated in clinical trials as therapeutic options for AML. Among them are sunitinib (SU11248) (**4**), which showed promising results in phase I/II clinical trials; sorafenib (Bay 43-9006) (**5**), which is limited to combination therapy; and crenolanib (CP-868-596) (**6**), which has demonstrated promising clinical activity both as a single agent and in combination therapy in AML patients (Figure 4). **4** and **5** are first-generation inhibitors already approved for the treatment of other diseases, whereas crenolanib, a second-generation inhibitor, was specifically developed for AML.^26,37–40^

**Figure 4:**
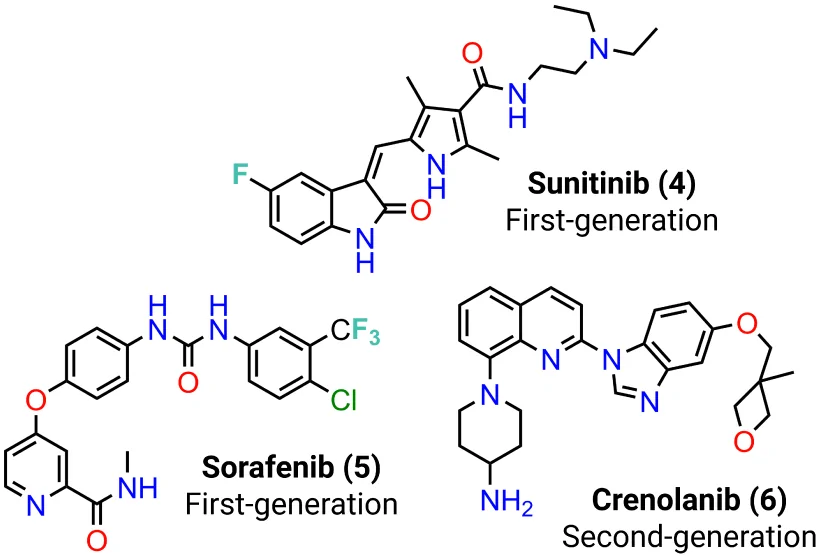
Additional FLT3 inhibitors under investigation.

FLT3i can be alternatively classified into type I inhibitors, such as midostaurin (**1**), gilteritinib (**2**), sunitinib (**4**), and crenolanib (**6**); and type II inhibitors, including sorafenib (**5**) and quizartinib (**3**). Both type I and type II inhibitors explore the hinge region as an anchoring point but can also explore adjacent hydrophobic pockets. Moreover, type I inhibitors exhibit high affinity for the DFG-in conformation of FLT3, whereas type II inhibitors bind to the DFG-out inactive conformation of FLT3. A conserved triad of amino acids located in the activation loop (A-loop), known as the DFG motif, which consists of the sequence aspartate (D), phenylalanine (F), and glycine (G), is present across all protein kinases. The FLT3 receptor can adopt either an active or inactive conformation depending on the orientation of these three residues. In the DFG-in conformation, the side chain of D829 is oriented toward the ATP-binding site (hinge), while the aromatic ring of the F830 side chain is oriented in the opposite direction. In turn, in the DFG-out conformation, a rearrangement of the activation loop occurs, in which the orientation of these residues is inverted, the aromatic ring of the F830 side chain is directed toward the hinge, and the side chain of D829 is oriented away from it. This structural rearrangement leads to the exposure of an additional pocket that is absent in the active DFG-in conformation. Type II inhibitors are able to occupy this additional region. Although type I inhibitors preferentially bind to the DFG-in conformation, they may also bind to the DFG-out conformation; however, in this case, they primarily interact with the hinge region and do not exploit the additional cavity accessed by type II inhibitors (Figure 5).^1,8,41,42^

**Figure 5:**
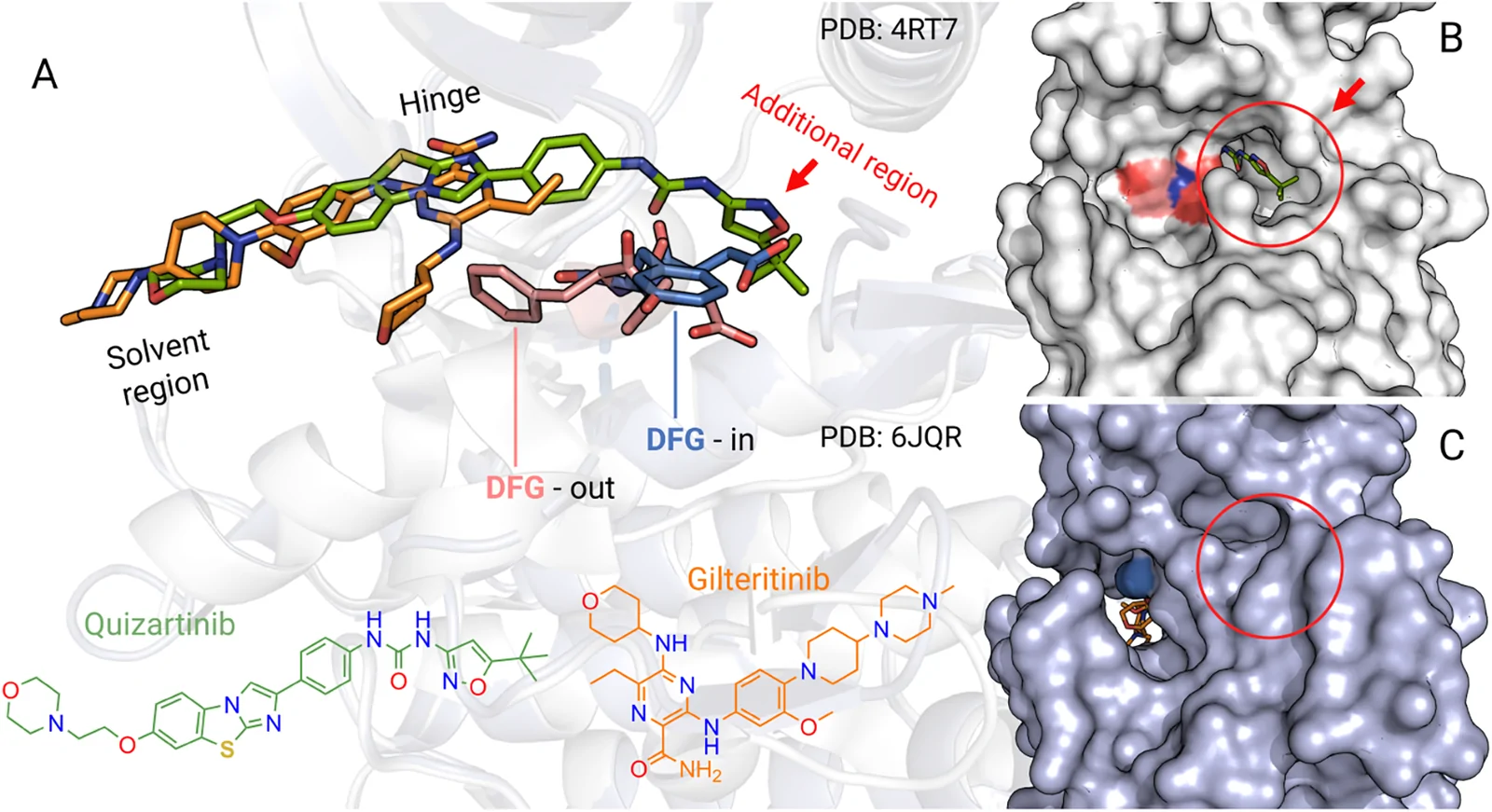
(**A**) Overlay of FLT3 structures deposited in the Protein Data Bank (PDB), showing the binding of the type II inhibitor quizartinib (**3**) (green-colored carbon atoms, PDB: 4RT7, DFG-out shown in salmon) and the type I inhibitor gilteritinib (**2**) (orange-colored carbon atoms, PDB: 6JQR, DFG-in shown in dark blue). (**B**) **3** extends into an additional cavity accessible in the inactive DFG-out conformation of FLT3. (**C**) whereas **2** occupies the ATP-binding site preferentially in the active DFG-in conformation. Structural visualization and inspection were performed using PyMOL.

Since type II inhibitors are distinguished by their ability to access regions not available in the DFG-in conformation, and because the amino acids in these regions are less conserved than those in the ATP-binding site, they tend to engage in specific interactions that confer a more selective profile against the target and several mutated forms. Quizartinib (**3**) exemplifies this concept, as a type II inhibitor that demonstrates higher activity in FLT3-ITD-positive cells, suggesting that interactions with less conserved regions may favor selectivity toward specific mutant forms.^7–9,43,44^

FLT3i, whether used alone or in combination with other therapies, can alleviate AML symptoms and improve patients’ overall survival. Drugs such as gilteritinib (**2**) and quizartinib (**3**) demonstrate excellent response rates in patients with FLT3-mutated AML, however, the duration of response is limited. Despite significant advances in the development of various FLT3i, treatment resistance remains a challenge, often resulting in short-lived responses.^7,45,46^

## Mechanisms of Resistance to FLT3 Inhibitors

Resistance to FLT3 inhibitors in AML patients can occur through on-target or off-target mechanisms.^46,47^ Point mutations in the tyrosine kinase domain (TKD) are responsible for on-target resistance. These mutations typically occur at residues within the activation loop, such as D835, and in the gatekeeper region, such as F691L, but can also involve other TKD residues, including I836, D839, and Y842, among others. Mutations at residue D835, located in the activation loop of the FLT3 receptor, reduce the efficacy of type II inhibitors, such as sorafenib (**5**) and quizartinib (**3**), since this mutation induces the DFG-in conformation. In contrast, type I inhibitors usually remain active against mutations at residue D835. On the other hand, the F691L mutation confers resistance not only to **5** and **3** but also to type I inhibitors, since this mutation does not directly affect the kinase conformation but alters the structural environment of the inhibitor-binding site, impacting both type I and type II inhibitors.^7,45,48–52^

The substitution of the aromatic residue phenylalanine (F) with a more flexible aliphatic residue, such as leucine (L), leads to the loss of favorable π interactions and may induce changes in the steric volume of the region, thereby reducing the accommodation of FLT3i.^7,45,48–52^

Mutations occurring at the gatekeeper residue represent a recurrent mechanism of acquired resistance in several oncogenic kinases, including FLT3-ITD (F691L), BCR-ABL (T315I), EGFR (T790M), PDGFRα (T674I), and ALK (L1196M).^49,53–56^ These residues control access to a hydrophobic region adjacent to the ATP-binding site (hydrophobic region I), which is not occupied by the nucleotide in its native binding mode but is frequently exploited by kinase inhibitors. In the case of the T790M mutation in EGFR, it has been demonstrated that resistance is associated with the restoration of the kinase’s affinity for ATP, thereby reducing the efficacy of competitive inhibitors. This behavior can be rationalized by the fact that mutation-induced structural changes preferentially affect the binding of inhibitors that explore regions adjacent to the hinge.^54^

For FLT3, the gatekeeper is a phenylalanine, which is larger and more hydrophobic than the threonine found in EGFR. The F691L mutation, which substitutes phenylalanine with leucine, alters the local conformational environment by promoting the repositioning of key residues within the binding site. This effect is particularly relevant for quizartinib (**3**), whose binding affinity depends on direct interactions with residue F691, as demonstrated by crystallographic studies (Figure 6). The F691L mutation has been associated with a substantial reduction in ligand binding affinity and, consequently, with resistance.^8,49^

**Figure 6:**
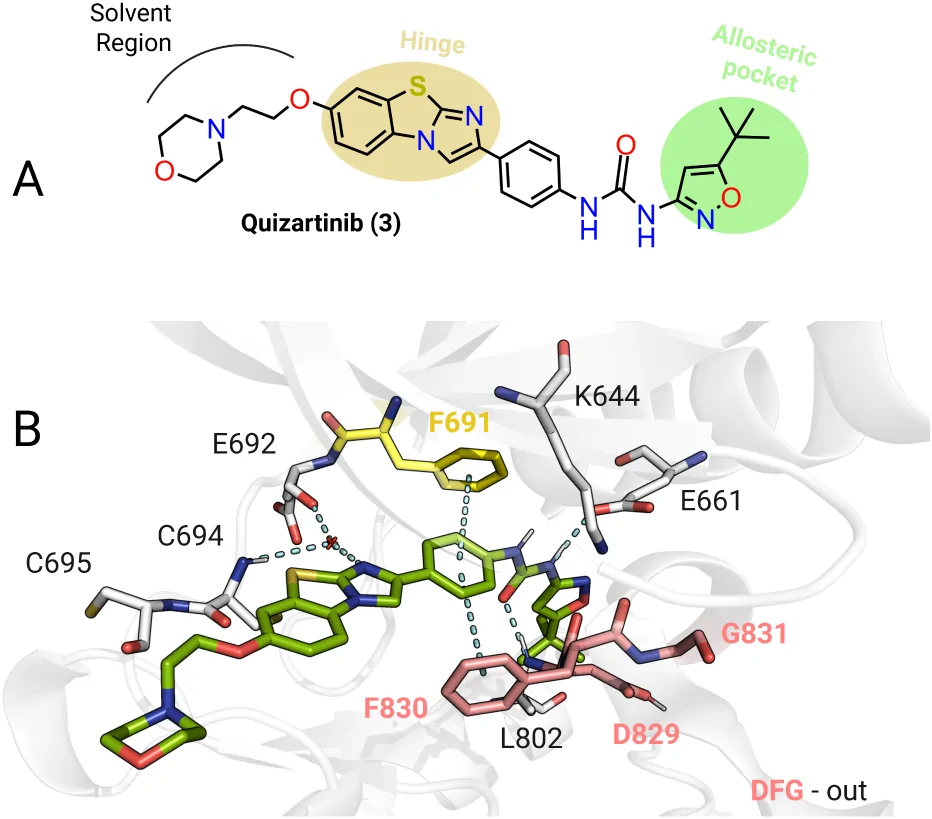
(**A**) Chemical structure of quizartinib (**3**). (**B**) Co-crystal structure of **3** (green-colored carbon atoms) bound to FLT3 (PDB: 4RT7), with key residues highlighted, including the gatekeeper residue F691, colored in yellow. Interactions between the ligand and the target, including water-mediated interactions, are indicated by light blue dashed lines between heavy atoms. Structural visualization and inspection were performed using PyMOL.

The central phenyl ring of quizartinib (**3**) establishes aromatic interactions with residues F691 (5.1 Å) and F830 (5.4 Å) of the DFG motif, which are essential for the stability of the complex. Alterations in either of these residues, such as those resulting from the F691L mutation, compromise the ligand-target interaction. This structural arrangement also explains why mutations associated with clinical resistance to **3** are predominantly concentrated at F691 and D835, the latter being a regulatory residue of the activation loop that directly influences the conformational orientation of the DFG motif.^8,49,57,58^

The imidazobenzothiazole core of compound **3** is positioned within the adenine-binding region, while the terminal *t*-butyl-isoxazole moiety occupies the allosteric cavity. This region is delineated by hydrophobic side chains, including residue L802, which marks the distal boundary of the pocket. The urea linker between the phenyl and isoxazole rings forms two hydrogen bonds, one with residue E661 (2.8 Å) of the αC-helix and another with the backbone amide of D829 (2.7 Å) from the DFG motif, establishing an interaction pattern recurrent among type II kinase inhibitors containing urea and amide. FLT3i generally possess an adenine-like moiety that fits into the cleft between the N- and C-lobes of the kinase domain and forms direct hydrogen bonds with the hinge region. **3**, however, lacks a group with these characteristics and therefore does not directly interact with the hinge. Instead, the imidazobenzothiazole moiety recruits a water molecule that acts as a bridge and establishes two hydrogen bonds with the polar residues C694 (3.4 Å) and E692 (2.2 Å).^8,49,58,59^

Beyond resistance mechanisms directly attributed to mutated forms of FLT3, off-target resistance mechanisms include the activation of pro-growth and pro-survival signaling pathways. These pathways can be activated either directly by the mutated FLT3 receptor or indirectly, in response to its inhibition, through alternative tyrosine kinases such as AXL and SYK. In addition, compensatory processes contribute to resistance, such as increased expression of the FLT3 ligand (FL) and the formation of a protective bone marrow microenvironment. One example is the activation of the Ras pathway, mediated by the secretion of fibroblast growth factor 2 (FGF2) by stromal cells. A study investigated the impact of the bone marrow microenvironment on the efficacy of FLT3 inhibitors. Traer and colleagues demonstrated that FGF2, secreted by stromal cells, protects FLT3-ITD+ leukemic cells from quizartinib-induced apoptosis. This protective effect is associated with the activation of signaling pathways that promote cell survival, contributing to treatment resistance.^9,24,46,60^

### Strategies to Overcome Resistance

The development of targeted therapies for the treatment of AML has emerged as a promising approach. Activating mutations in the FLT3 receptor are frequently associated with a more aggressive disease profile. The combination of FLT3 inhibitors with other classes of drugs has been extensively investigated to overcome resistance and achieve durable remissions. In addition, several FLT3i are currently in active development, both in preclinical and clinical stages, aiming to overcome on-target resistance. Among these are multitarget inhibitors approved for malignancies unrelated to AML, as well as next-generation, more specific agents.^6,24,61–64^

### Combination Therapy as Therapeutic Alternative for AML

The use of FLT3i as monotherapy for AML often results in drug resistance, short and transient responses, and unsatisfactory clinical outcomes due to pathological alterations. These alterations include, among others, the compensatory activation of downstream signaling pathways of FLT3. In this context, the combination of FLT3i with other therapies has been extensively investigated, suggesting that simultaneous blockade of synergistic signaling pathways may enhance treatment efficacy and help overcome resistance.^24,61^

Pim kinase inhibitors have demonstrated synergistic cytotoxicity when combined with FLT3 inhibitors. Overexpression of Pim-1, in particular, reduces sensitivity to FLT3i, impairing their apoptotic effects and contributing to treatment resistance. Studies have shown that coadministration of Pim inhibitors induces stronger apoptosis in FLT3-mutated cells. For instance, the combination of the Pim inhibitor AZD1208 (**7**; Figure 7) with an FLT3i has proven effective in inducing cell cycle arrest and apoptosis in FLT3-ITD AML cells in vitro, as well as in reducing tumor progression in xenograft mouse models engrafted with MV4-11 cells. This effect was associated with increased proteasomal degradation of MCL-1. Other agents, such as the Pim inhibitor SEL24 (**8**; Figure 7), are also reported in the literature.^65–72^

**Figure 7:**
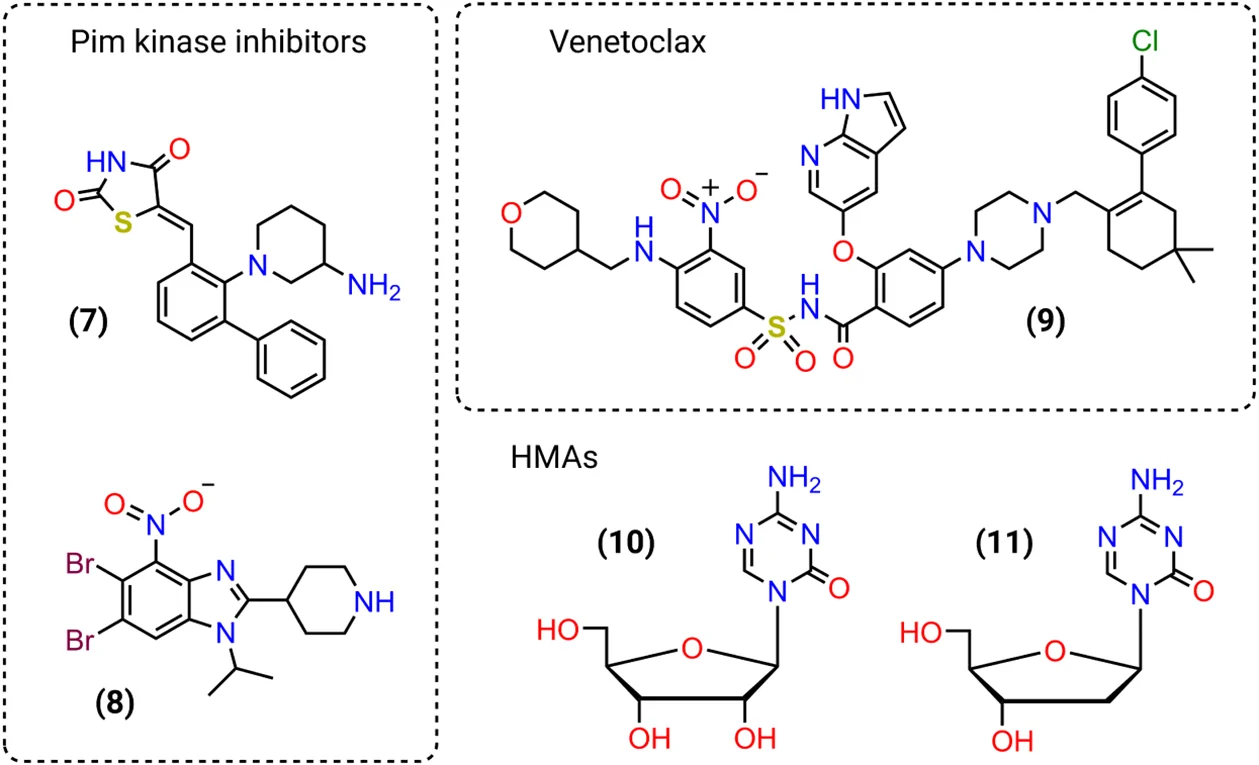
Potential agents for use in combination therapy with FLT3i for AML.

Preclinical studies have shown that FLT3-ITD-mutated blasts exhibit higher BCL-2 expression compared to FLT3 wild-type blasts, and that the overexpression of antiapoptotic proteins constitutes an off-target resistance mechanism to FLT3 inhibitors.^73,74^ Venetoclax (**9**; Figure 7) has demonstrated synergistic antileukemic activity when combined with midostaurin (**1**), gilteritinib (**2**), and quizartinib (**3**). The combination of FLT3i with agents that potently induce apoptosis has the potential to achieve more durable responses in FLT3-ITD+ patients.^75–78^

Histone deacetylase inhibitors (HDACi) exhibit significant activity against hematologic malignancies, acting as epigenetic antineoplastic agents. HDACi have been shown to overcome resistance to FLT3 inhibitors in AML cells, and the synergistic antitumor effects of combining FLT3i with HDACi in FLT3-ITD^+^ cells have been widely demonstrated. Inhibition of FLT3 can lead to upregulation of HDAC-8 through the activation of transcription factors FOXO1 (Forkhead box protein O1) and FOXO3 (Forkhead box protein O3). In one study, HDAC-8 was found to be overexpressed in MV4-11 and MOLM-13 cells following quizartinib (**3**) treatment.^79,80^

Futhermore, the combination of FLT3 inhibitors with hypomethylating agents (HMAs) such as azacitidine (**10**; Figure 7) or decitabine (**11**; Figure 7) has proven to be a promising therapeutic strategy. This association is attractive because it combines synergistic cytotoxic activity, observed in experimental studies, with the good clinical tolerability of HMAs, especially in elderly patients or those ineligible for intensive chemotherapy. Hypomethylating agents contribute to modulating the bone marrow microenvironment and sensitizing leukemic cells to FLT3 inhibition, thereby promoting more sustained responses.^81–85^

### Application of Multitarget Inhibitors to Overcome Resistance

Although combinations of FLT3 inhibitors with other therapies have shown positive efficacy, unfavorable pharmacokinetic profiles, drug–drug interactions, and poor patient adherence may hinder the progress of these combined approaches. Such combinations are largely designed to suppress off-target resistance mechanisms, such as the compensatory activation of signaling pathways triggered in response to FLT3 inhibition. On the other hand, multitarget inhibitors have emerged as a rationally optimized strategy, integrating within a single molecule the ability to inhibit FLT3 while blocking associated escape pathways, thus simplifying the therapeutic regimen. These multitarget inhibitors have proven effective not only against off-target resistance mechanisms but also against on-target resistance, arising from mutations within the FLT3 itself, establishing them as a promising alternative to conventional combination therapies. Moreover, the chemical structures of these agents may inspire researchers to design more effective anti-AML drugs in the future.^33,49,86–95^

By acting simultaneously on two or more targets, multitarget inhibitors have shown greater potential to overcome compensatory mechanisms activated in response to isolated FLT3 inhibition. One example is gilteritinib (**2**), a drug already approved for the treatment of AML, described as an effective dual FLT3 and AXL receptor tyrosine kinase inhibitor. Studies have shown that treatment with midostaurin (**1**) or quizartinib (**3**) leads to a significant increase in AXL phosphorylation in FLT3-ITD-positive cells, which contributes to resistance to these drugs. In this context, simultaneous inhibition has proven effective in suppressing this adaptive escape mechanism. In BaF3 cells, **2** also exhibited inhibitory activity against FLT3-ITD (IC_50_: 1.8 nM) and FLT3-ITD-D835Y (IC_50_: 2.1 nM), indicating its effectiveness in inhibiting both the mutant form and resistance-associated variants. Thus, **2** represents a practical example for the development of multitarget inhibitors with enhanced therapeutic efficiency.^33,87^

Another example is pacritinib (SB1518) (**12**), a dual FLT3 and JAK2 (Janus kinase 2) inhibitor. Aberrant activation of JAK2 has been associated with various hematologic malignancies, and experimental data have shown that JAK2 levels increase following treatment with FLT3 inhibitors, contributing to therapeutic resistance. Therefore, the concomitant inhibition of FLT3 and JAK2 represents a promising strategy. Although **12** exhibits nanomolar-scale inhibitory activity against FLT3, its efficacy is more pronounced toward the nonmutated form of the enzyme, and the compound is not approved for AML treatment. Nevertheless, by combining within a single structure the ability to block both FLT3 and JAK2, **12** stands out as a relevant structural model, serving as a starting point for the development of new, more selective and potent compounds capable of acting more comprehensively against both on-target and off-target resistance mechanisms.^88^

The multitarget approach can be useful to overcome the challenges imposed by off-target resistance mechanisms by preventing the activation of compensatory signaling pathways. Although gilteritinib (**2**) is a FLT3 and AXL inhibitor, which is particularly advantageous in the context of AXL-mediated resistance in AML, it is important to note that its activity is not limited to these targets. Studies have shown that **2** also inhibits other tyrosine kinases, such as LTK (leukocyte receptor tyrosine kinase) and ALK (anaplastic lymphoma kinase). This multitarget property may be beneficial in specific therapeutic contexts, especially when signaling crosstalk or compensatory pathways are involved in tumor progression. However, the inhibition of multiple targets may also compromise drug selectivity and safety.^33^

Thus, although the ability to efficiently and simultaneously inhibit FLT3 and AXL represents an important advantage of gilteritinib (**2**), its activity against other kinases should be carefully considered. In this regard, **2** clearly illustrates the common dilemma in the development of multitarget inhibitors, balancing enhanced therapeutic efficacy with the maintenance of an acceptable safety profile. Yuan and colleagues highlight the synergistic potential of targeting FLT3 kinase in combination with other relevant therapeutic targets in cancer treatment, providing a comprehensive overview of the most recent scientific advances.^95^

In the crystal structure of the FLT3-gilteritinib complex (PDB: 6JQR), the amide group of gilteritinib (**2**) forms hydrogen bonds with residues located in the hinge region. The carbonyl oxygen of the amide acts as a hydrogen bond acceptor, interacting with C694 (2.8 Å), while the adjacent amino group functions as a hydrogen bond donor, forming a bond with E692 (2.8 Å) (Figure 8B).^58,59,96^

**Figure 8:**
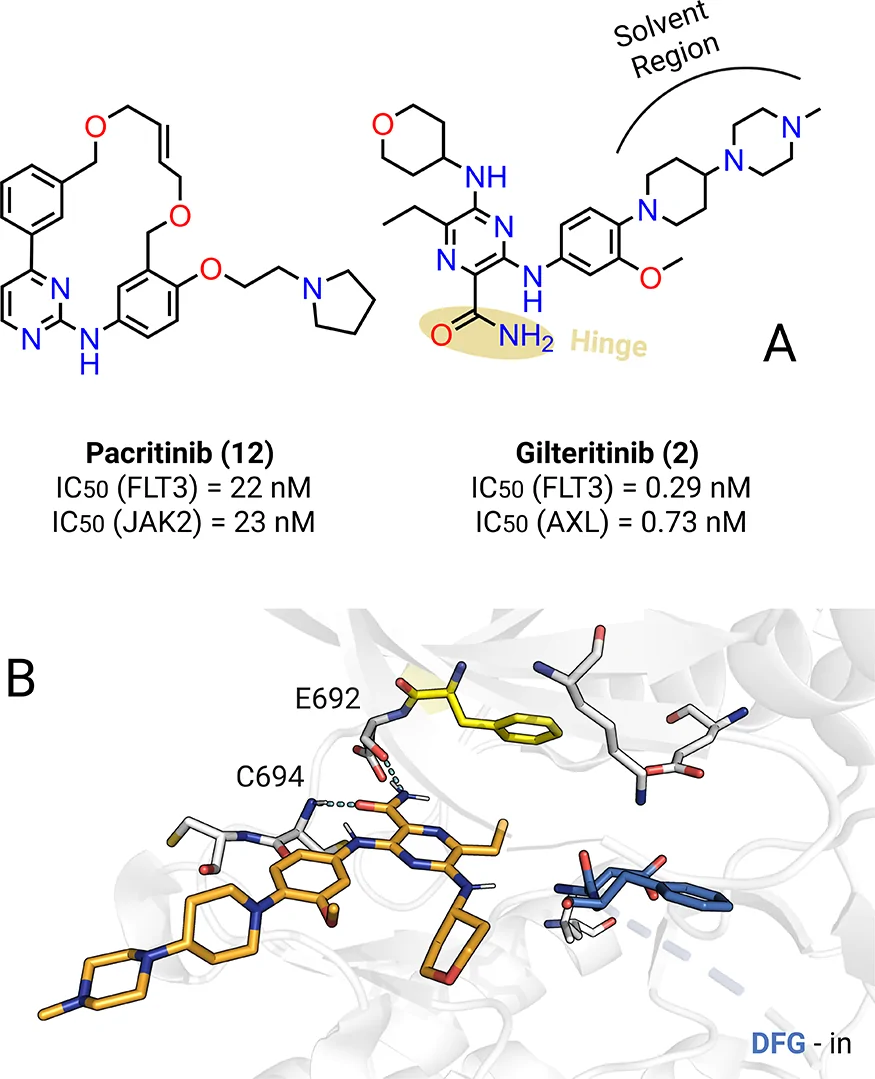
(**A**) Chemical structures of compounds pacritinib (**12**, left) and gilteritinib (**2**, right). (**B**) Co-crystal structure of **2** (orange-colored carbon atoms) bound to FLT3 (PDB: 6JQR), with key residues highlighted. Interactions between the ligand and the target are indicated by light blue dashed lines between heavy atoms. Structural visualization and inspection were performed using PyMOL.

It is important to note that many compounds described as “dual inhibitors” in the literature are, in practice, multitarget inhibitors, exhibiting significant activity against more than two targets. Frequently, authors emphasize only the most relevant targets for the pathology under study, which can lead to a simplified perception of the selectivity of these molecules. The compound **13**, similarly to pacritinib (**12**), demonstrated activity against FLT3 and JAK2. However, **13** also exhibited strong activity against JAK3 (Janus kinase 3). To improve its selectivity, structural modifications were made based on the mode of interaction with JAK2 and JAK3. The introduction of a bulky group in **13** reduced its activity against JAK3 while maintaining selectivity toward JAK2, resulting in the derivative **14**. Although compound **14** still requires improvements in its properties, this example reinforces the relevance of applying rational structural modifications to refine the selectivity profile of multitarget ligands (Figure 9).^92^

**Figure 9:**
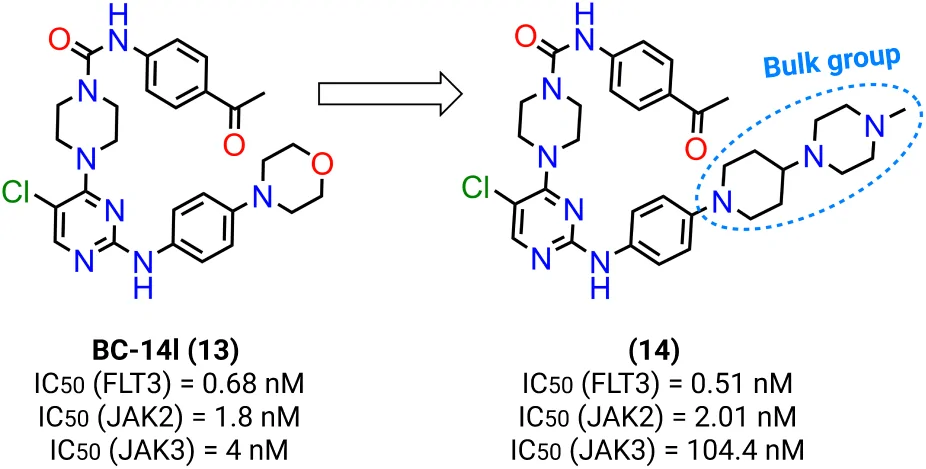
Structural modification of the BC-14I (**13**) inhibitor aimed at reducing activity against JAK3 while maintaining potency toward FLT3 and JAK2.

Some drugs have shown good activity against FLT3 mutations, demonstrating potential in cell-based assays against the lineage expressing the resistance mutation F691L. Pexidartinib (PLX3397) (**15**), a triple kinase inhibitor (inhibition of FLT3, CSF1R—colony stimulating factor 1 receptor, and c-KIT—KIT proto-oncogene receptor tyrosine kinase), has been investigated in several solid tumors and has also shown efficacy against FLT3 mutations (Figure 10). According to Smith et al., **15** effectively inhibited FLT3 signaling in cells harboring the FLT3-ITD mutation, with nanomolar-scale activity, including an enzymatic assay targeting the FLT3-ITD mutant, with IC_50_ values in the same range. In contrast, its efficacy was considerably reduced in cells containing only wild-type FLT3, displaying micromolar-scale activity. These results are particularly relevant because they highlight pexidartinib’s preference for binding to the mutant FLT3-ITD form, suggesting that the compound may represent a more selective and effective therapeutic approach against tumors associated with this mutation, while minimizing effects on healthy cells expressing native FLT3. Furthermore, **15** has also demonstrated the ability to inhibit the F691L isoform in BaF3 cells, reinforcing its potential as a promising agent for the treatment of leukemias associated with FLT3 mutations, including those that exhibit acquired resistance to other inhibitors, such as quizartinib (**3**). Like most type II inhibitors, **15** showed vulnerability to D835 mutations.^49^

**Figure 10:**
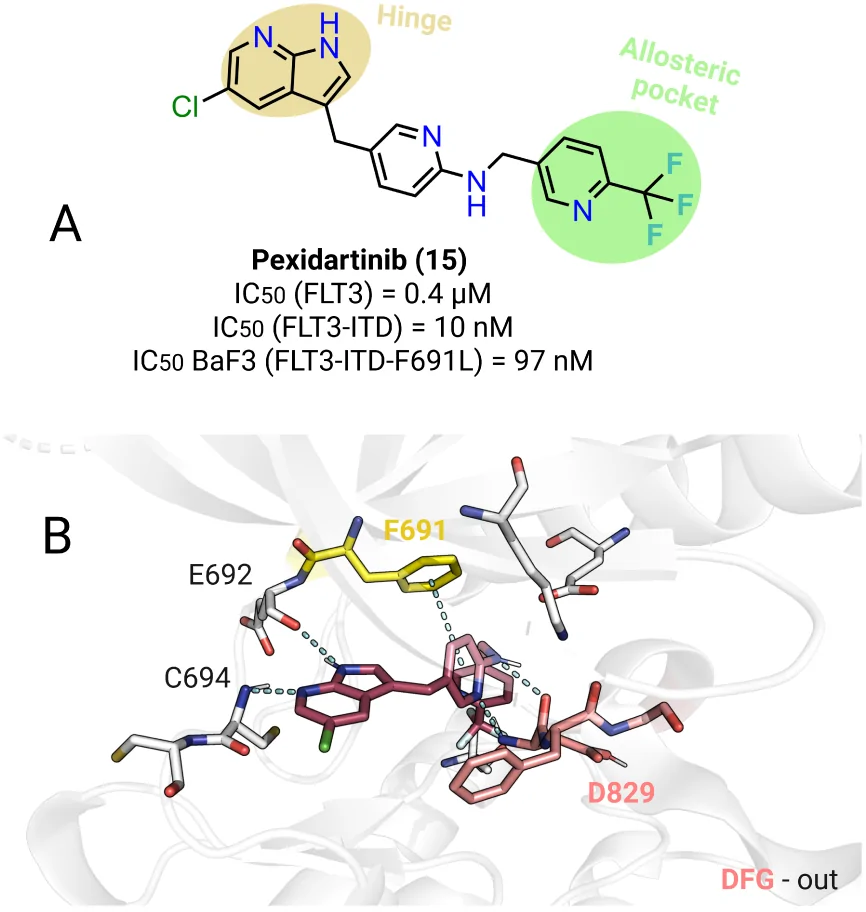
(**A**) Chemical structure of compound pexidartinib (**15**). (**B**) Docking of **15** (dark pink-colored carbon atoms) into the FLT3 kinase (PDB: 4RT7), performed using the GOLD program (ChemPLP: 106.84). The ChemPLP scoring function was selected based on a root-mean-square deviation value (RMSD: 1.30 Å) lower than the crystal resolution. Key residues are highlighted. Interactions between the ligand and the target are indicated by light blue dashed lines between heavy atoms. Structural visualization and inspection were performed using PyMOL.

Since cocrystallized structures of this and other inhibitors of interest with FLT3 are not yet available, docking analyses of **15** and additional examples described herein were conducted in GOLD 5.8.1 program for visual inspection and appropriate discussion of the expected ligand-protein interactions. Molecular docking calculations were carried out using the GOLD genetic algorithm, performing 10 independent runs for each ligand and employing the FLT3 crystal structure with PDB ID: 4RT7. The docking protocol was validated by redocking, and ChemPLP was selected as the scoring function based on a root-mean-square deviation (RMSD) value of 1.30 Å, which is lower than the crystallographic resolution.

The docking pose of pexidartinib (**15**), as well as the interaction mode of cocrystallized quizartinib (**3**), indicates that both compounds bind to the ATP adenine-binding region, showing a propensity to establish hydrogen bonds with residues C694 (2.9 Å) and E692 (3.3 Å). A relevant structural difference between **3** and **15** lies in the absence of the urea moiety in **15**. While **3** contains a urea group between the phenyl and isoxazole rings, which is responsible for interactions with residue E661 of the αC-helix and with the amino group of the D829 backbone, **15** features a secondary amine in this region. Due to resonance-driven delocalization of the nitrogen lone pair, this amine exhibits a greater tendency to act as a hydrogen bond donor, potentially establishing contact with the backbone carbonyl oxygen of D829 (2.9 Å). Additionally, pexidartinib was found to be favorable for aromatic interaction with F691 (5.2 Å), while the nitrogen atom present in the central heterocycle may act as a hydrogen bond acceptor with the backbone amino group of the DFG aspartate (3.1 Å).^49,58,59^

Other multitarget inhibitors cited in the literature include cabozantinib (XL184) (**16**; Figure 11) and ponatinib (AP24534) (**17**; Figure 12). **16** is a multitarget inhibitor that acts on tyrosine kinase receptors FLT3, AXL, MET, VEGFR, and c-KIT. Approved for the treatment of medullary thyroid carcinoma and renal cell carcinoma, this compound has demonstrated positive clinical efficacy in patients with FLT3-ITD mutated AML. Studies have shown that **16** inhibits FLT3 in cells harboring the ITD mutation and suppresses FLT3 phosphorylation in BaF3 cells with the F691L mutation (IC_50_: 2.86 nM).^91^ The pan-BCR-ABL inhibitor **17**, approved for the treatment of chronic myeloid leukemia, also exhibits inhibitory activity against FLT3. **17** potently inhibited the proliferation of cells expressing FLT3-ITD and maintained activity against the resistance-conferring F691L mutation in BaF3 cells (IC_50_: 52 nM). As expected, substitutions at residue D835 in the activation loop of the FLT3 receptor conferred resistance to both inhibitors.^91,93,94^

**Figure 11:**
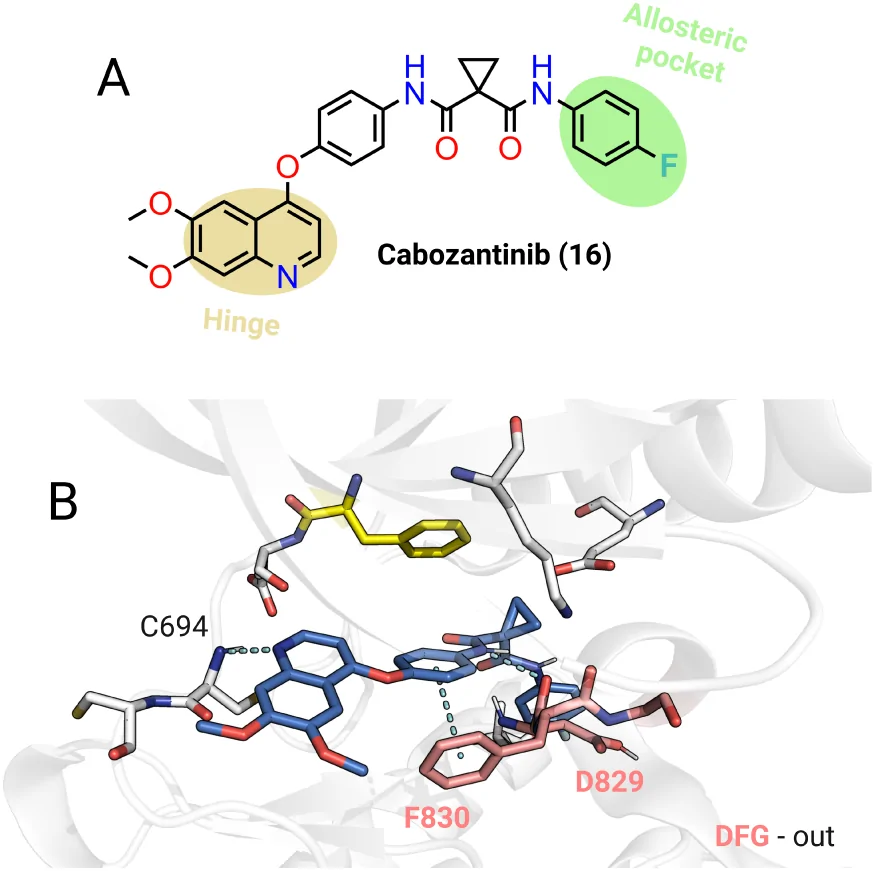
(**A**) Chemical structure of compound cabozantinib (**16**). (**B**) Docking of **16** (blue-colored carbon atoms) into FLT3 kinase (PDB: 4RT7) performed using the GOLD program (CHMPLP: 100.61), under the same conditions described for **15**.

**Figure 12:**
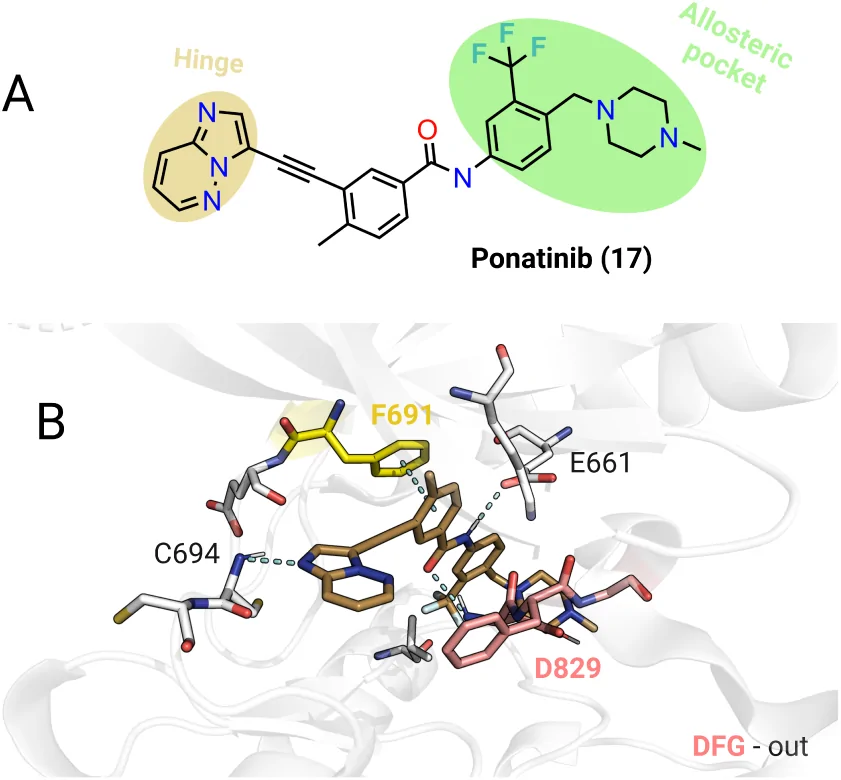
(**A**) Chemical structure of compound ponatinib (**17**). (**B**) Docking of **17** (brown-colored carbon atoms) into FLT3 kinase (PDB: 4RT7) performed using the GOLD program (CHMPLP: 110.58), under the same conditions described for **15**.

In the docking binding model, cabozantinib (**16**) forms hydrogen bonds with residue C694 (3.0 Å), located in the hinge region, and with residue D829 (3.0 Å) of the DFG motif. Similarly, ponatinib (**17**) interacts with C694 (3.0 Å). Due to resonance effects, the lone pair of the amide nitrogen in **17** shows a greater tendency to act as a hydrogen bond donor rather than an acceptor, forming an interaction with the carbonyl oxygen of E661 (3.1 Å). Simultaneously, the backbone amino group of D829 (2.7 Å) donates a hydrogen to the carbonyl oxygen of ponatinib’s amide, contributing to the stabilization of the complex in the inactive conformation of the kinase. Additionally, both compounds were found to be favorable for establishing hydrophobic aromatic interactions, with cabozantinib interacting with F830 (4.6 Å) and ponatinib with F691 (3.7 Å).^58,59^

Originally developed as a BTK (Bruton’s tyrosine kinase) inhibitor for the treatment of lymphoid malignancies, ibrutinib (PCI-32765) (**18**; Figure 13) exhibits a multitarget profile, with activity against other kinases, including FLT3 in BaF3 cells (GI_50_: 0.12 μM). This secondary activity inspired the development of new structurally derived compounds, such as CHMFL-FLT3-122 (**19**; Figure 13), a selective FLT3 inhibitor capable of suppressing the proliferation of BaF3-FLT3-ITD cells (GI_50_: 11 nM).^97^

**Figure 13:**
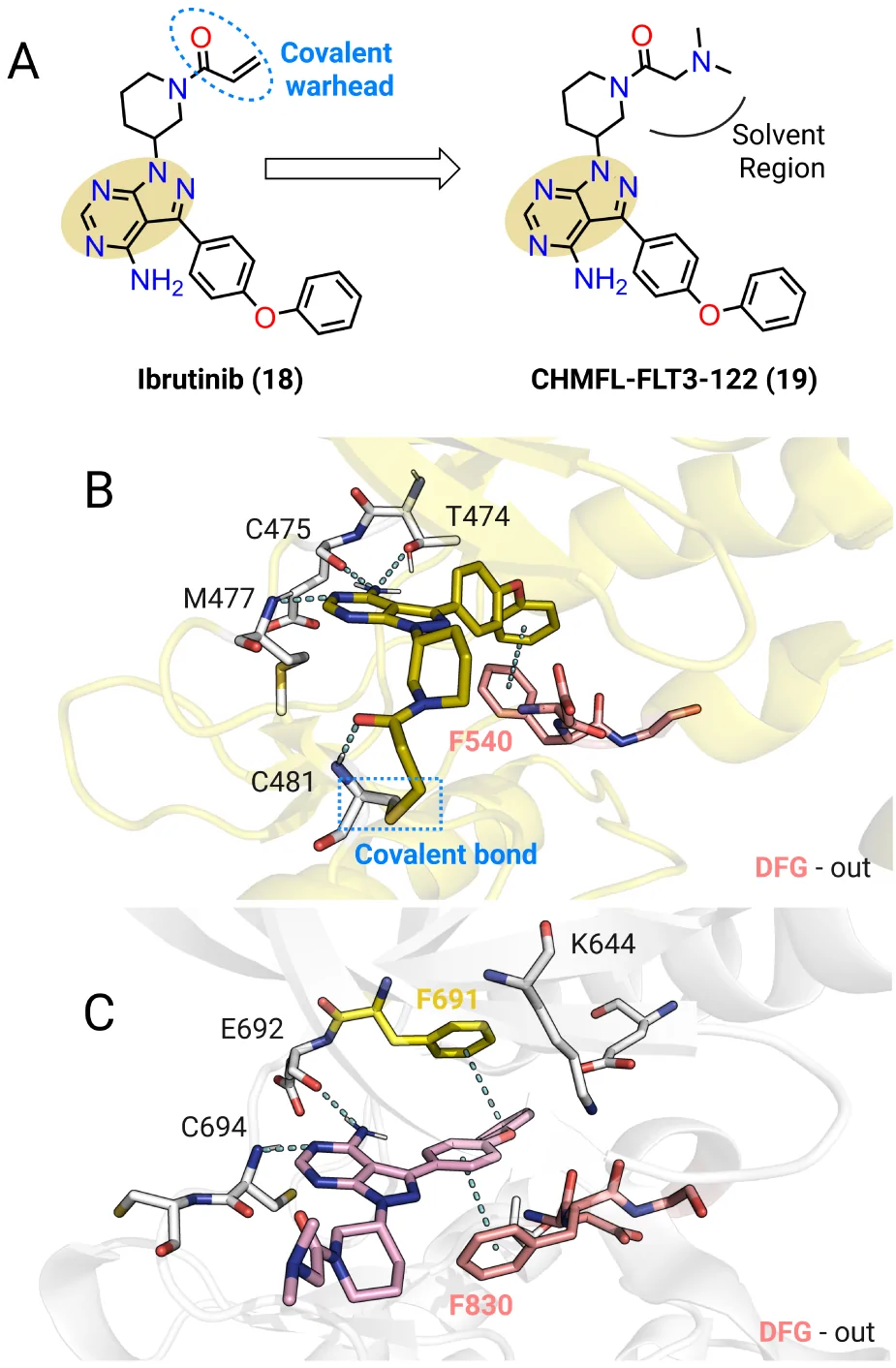
(**A**) Chemical structure of compound ibrutinib (**18**, left) and CHMFL-FLT3-122 (**19**, right). (**B**) Co-crystal structure of **18** (olive-colored carbon atoms) bound to BTK (PDB: 5P9J). (**C**) Docking of CHMFL-FLT3-122 (**19**) (pink-colored carbon atoms) into FLT3 kinase (PDB: 4RT7) performed using the GOLD program (CHMPLP: 91.08), under the same conditions described for **15**.

The identification of ibrutinib (**18**) as an inhibitor of FLT3 motivated its use as an initial scaffold for the development of more potent and selective structural analogues. Optimization was guided by the analysis of its binding mode to the kinases BTK and FLT3. **18** binds irreversibly to BTK through the formation of a covalent bond between its electrophilic acrylamide moiety and the C481 residue. In FLT3, however, this covalent interaction is not observed. Based on this difference, the moiety responsible for irreversible BTK binding was removed during the structural optimization process, culminating in the synthesis of CHMFL-FLT3-122 (**19**), resulting in reduced affinity for BTK, while concomitantly promoting increased potency against FLT3.^97^

The compound CHMFL-FLT3-122 (**19**) appears to establish a series of interactions with FLT3 (Figure 13C). The nitrogen-containing heterocyclic ring interacts with residue C694 (2.7 Å) in the hinge region, while the primary amino group attached to this heterocycle forms a hydrogen bond with E692 (3.2 Å), contributing to the anchoring of the compound within the ATP-binding site. Additionally, the diaryl ether fragment of **19** extends toward the adjacent hydrophobic region, where its proximal aromatic ring interacts with residue F830 (4.6 Å) of the DFG motif, and its terminal aromatic ring establishes interactions with the gatekeeper residue F691 (4.5 Å). It is also noteworthy that the ether oxygen atom, although a weak hydrogen bond acceptor due to resonance effects between the two aromatic rings, may potentially participate in geometry-dependent polar interactions with positively charged residues under physiological conditions, such as K644 (3.4 Å).^58,59,97^

Not all compounds discussed in this work have crystallographic structures in complex with FLT3. However, structural data are available for some of these inhibitors in complex with other kinases, such as c-KIT, which, like FLT3, belongs to class III receptor tyrosine kinases and shares similar structural features (Figure 14). It is unlikely that the binding modes of these ligands differ substantially between these proteins, which allows for a visual comparison between the docking poses obtained for FLT3 and experimentally determined binding modes in related kinases. In this context, crystal structures of pexidartinib (**15**, PDB: 7KHG) and ponatinib (**17**, PDB: 4U0I) in complex with c-KIT provide a useful reference, and comparison with the corresponding docking poses in FLT3 suggests consistent binding orientations.^98,99^

**Figure 14:**
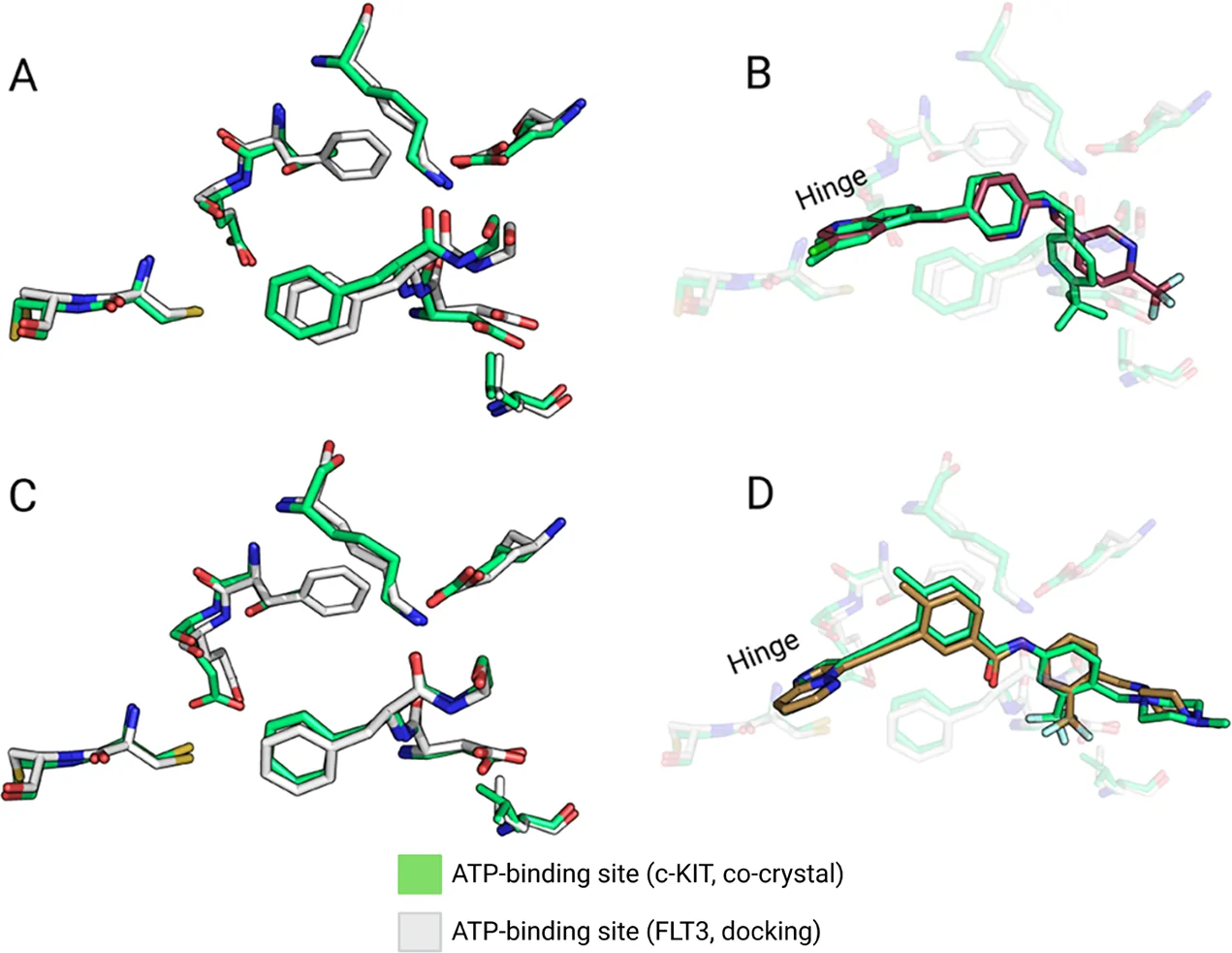
Structural comparison of binding modes in c-KIT and FLT3. (**A**) Superposition of key residues from the ATP-binding site of FLT3 (PDB: 4RT7) and c-KIT (PDB: 7KHG). (**B**) Superposition of pexidartinib (**15**) cocrystallized with c-KIT and the docking pose obtained in FLT3. (**C**) Superposition of key residues from the ATP-binding site of FLT3 (PDB: 4RT7) and c-KIT (PDB: 4U0I). (**D**) Superposition of ponatinib (**17**) cocrystallized with c-KIT and the docking pose obtained in FLT3.

### New-Generation FLT3 Inhibitors

As previously mentioned, FLT3 mutations are quite frequent and are associated with a poorer prognosis in AML. Although there is a substantial number of FLT3 inhibitors, the emergence of resistance-associated mutations during disease progression remains a challenge. To overcome these limitations, the so-called next-generation FLT3i have been developed with a focus on higher selectivity and the ability to inhibit FLT3-ITD-TKD mutations. These inhibitors aim to surpass the constraints of the previous generations.^6,100^

Among the next-generation inhibitors described in the literature, MZH29 (**20**; Figure 15) stands out as a compound developed in the context of efforts to overcome resistance mutations. Enzymatic and cellular assays have demonstrated that this compound exerts inhibitory effects on mutant FLT3, including the FLT3-ITD-F691L mutation, which is known to confer resistance to currently available inhibitors. In animal models, **20** induced tumor regression and prolonged survival with lower toxicity compared to quizartinib (**3**). These findings indicate that **20** has strong potential as a potent and selective FLT3 inhibitor, representing an advancement in the development of next-generation therapies for AML.^44^

**Figure 15:**
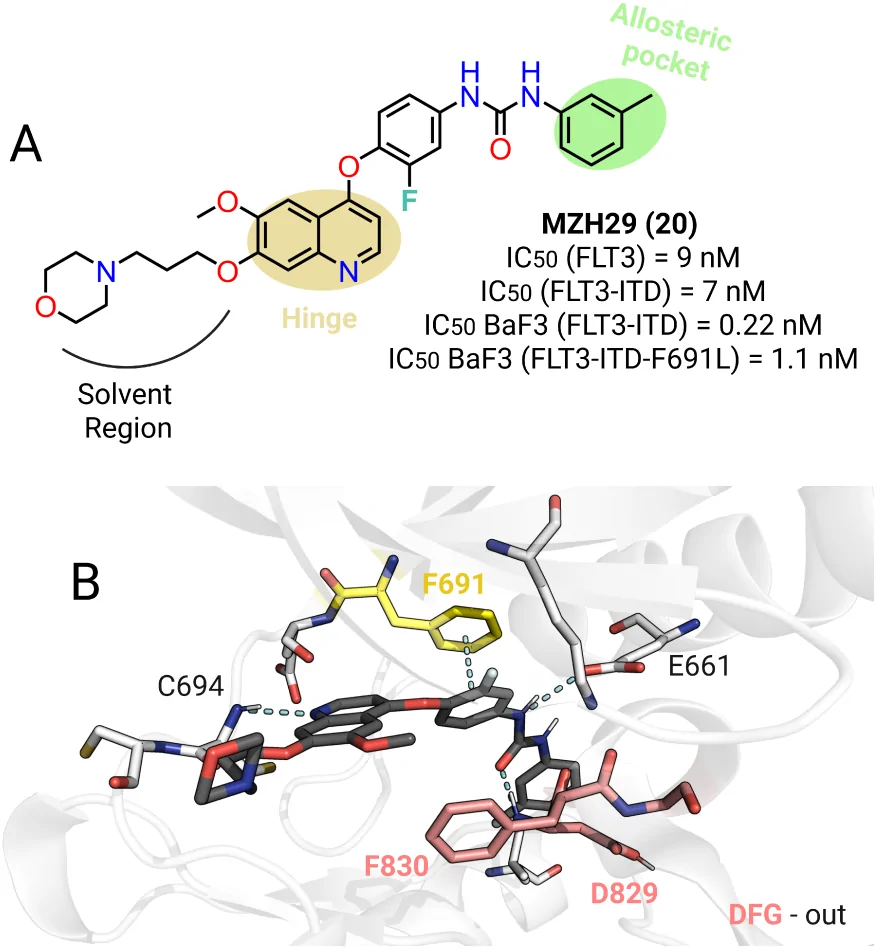
(**A**) Chemical structure of compound MZH29 (**20**). (**B**) Docking of **20** (black-colored carbon atoms) into FLT3 kinase (PDB: 4RT7) performed using the GOLD program (CHMPLP: 102.39), under the same conditions described for **15**.

MZH29 (**20**) exhibits structural similarity to already described type II FLT3i, featuring a heterocyclic ring that anchors in the hinge region and a urea linker connecting two aromatic rings, one central and one terminal, which potentially reaches the allosteric pocket in DFG-out conformation. The quinoline moiety of **20** forms an H-bond with residue C694 (3.0 Å), while molecular docking also indicated the potential formation of H-bonds with residues D829 (2.7 Å) and E661 (3.2 Å), as well as interaction with F691 (4.0 Å).^44,58,59^

Covalent inhibitors have recently emerged as a promising strategy to overcome drug resistance to protein kinase inhibition, as exemplified by successful agents such as ibrutinib (**18**). Although compounds like penicillin also act covalently, their discovery occurred serendipitously, and for a long time, the development of irreversible ligands was avoided due to concerns regarding potential toxicity and poor selectivity. With advances in rational drug design and the advent of targeted covalent inhibitors, interest in this class of molecules has grown considerably. In the context of FLT3, the first covalent inhibitor reported for AML treatment was FF-10101 (**21**; Figure 16). This compound contains an acrylamide group serving as a “warhead”, which reacts selectively with the C695 residue of FLT3 to form a stable covalent bond. This reactive moiety, typically a moderate electrophile, plays a critical role in defining the activity, selectivity, and biological profile of the ligand.^101–106^

**Figure 16:**
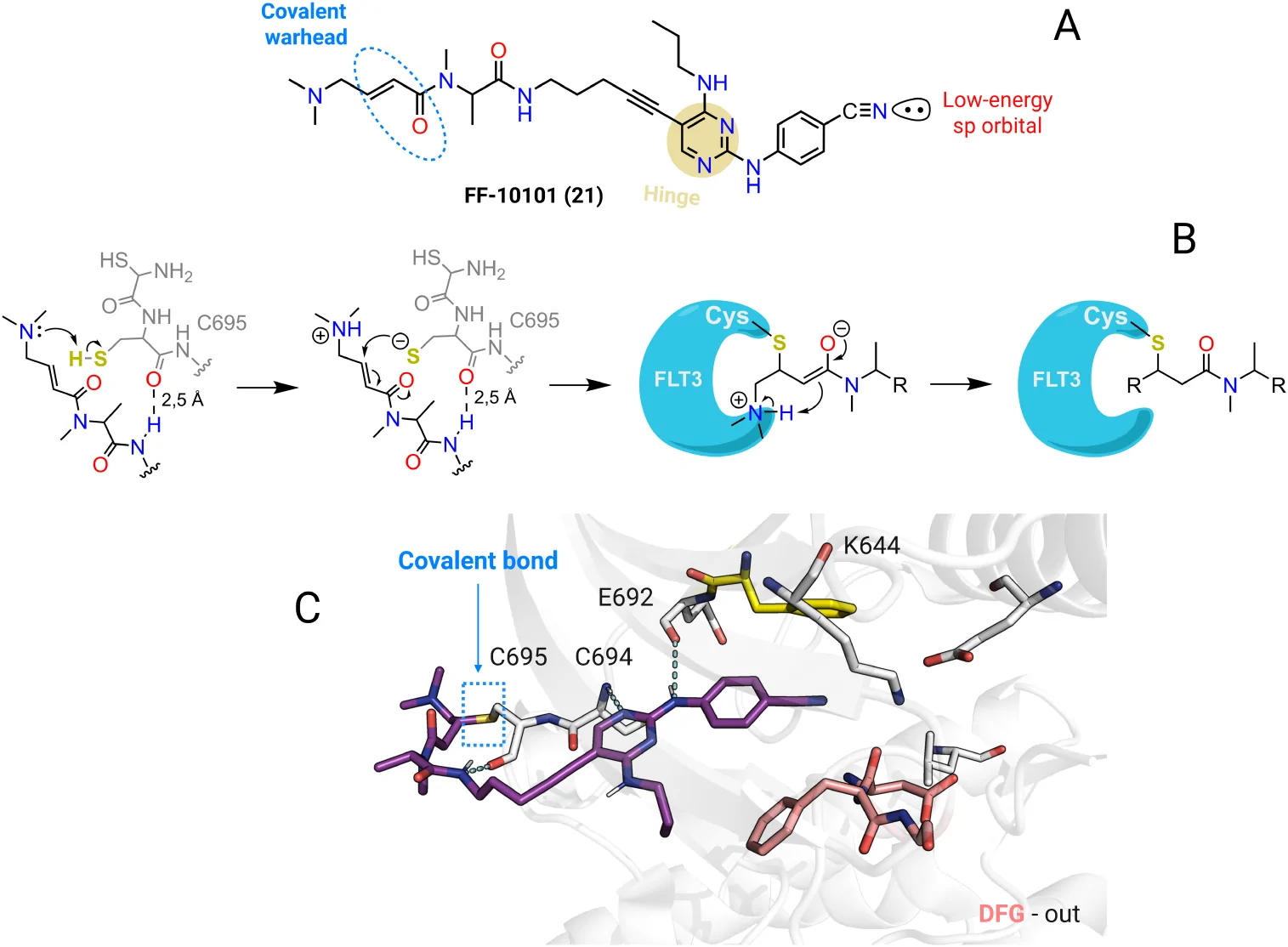
(**A**) Chemical structure of FF-10101 (**21**). (**B**) Covalent interaction of **21** with C695 of FLT3. Deprotonation of the cysteine may generate a reactive thiolate, which performs a nucleophilic attack on the β-carbon of the acrylamide (Michael acceptor), leading to the formation of the covalent adduct. (**C**) Co-crystal structure of **21** (purple-colored carbon atoms) bound to FLT3 (PDB: 5X02), with key residues highlighted. Interactions between the ligand and the target are indicated by light blue dashed lines between heavy atoms. Structural visualization and inspection were performed using PyMOL.

In vitro assays demonstrated that FF-10101 (**21**) exhibits activity against relevant FLT3 mutations. In human cell lines such as MV4-11, MOLM-13, and MOLM-14, **21** showed inhibitory effects against FLT3 internal tandem duplication (FLT3-ITD), with GI_50_ values of 0.83, 1.1, and 1.5 nM, respectively. Similar inhibitory activity was observed in 32D cells harboring FLT3-ITD (GI_50_: 1.9 nM), FLT3-ITD-D835Y (GI_50_: 0.81 nM), and FLT3-ITD-F691L (GI_50_: 10 nM). In subcutaneous implantation mouse models, orally administered **21** inhibited the growth of 32D cells expressing quizartinib-resistant mutations, such as F691L.^105^

The cocrystal structure of FLT3 with FF-10101 (**21**) (PDB: 5X02) reveals the formation of a covalent bond with residue C695 (Figure 16B), as expected. Additionally, a possible hydrogen bond with the same residue is observed at a distance of 2.5 Å. Compound **21** also interacts through hydrogen bonds with residues C694 (3.0 Å) and E692 (3.5 Å). It should be noted that nitriles are weakly basic and poorly nucleophilic, as the nitrogen lone pair resides in a low-energy *sp* orbital. Nevertheless, in the structural context of compound **21**, this nitrogen may potentially participate in a weak polar interaction, acting as a hydrogen bond acceptor, given that residue K644 (3.5 Å) is protonated under physiological conditions.^58,59,105^

Another FLT3 inhibitor that falls into the next-generation category is marbotinib (**22**; Figure 17), which acts selectively on FLT3 and is capable of inhibiting both FLT3-ITD and resistant variants. Beyer and colleagues demonstrated through molecular docking that **22** fits within both the active (DFG-in) and inactive (DFG-out) conformations of the FLT3 receptor adopting diverse and specific bioactive conformations, characterizing it as a hybrid inhibitor, the first of its kind to target this enzyme. When MV4-11 and BaF3 cells were treated with **22**, FLT3-ITD phosphorylation was inhibited. **22** was also effective against BaF3 cells expressing FLT3-ITD-D835Y (IC_50_: 17.9 nM), one of the most common mutations associated with resistance to quizartinib (**3**) and type II inhibitors in general. BaF3 cells expressing wild-type FLT3 remained unaffected under **22** treatment, confirming its selectivity toward the mutant forms.^107–109^

**Figure 17:**
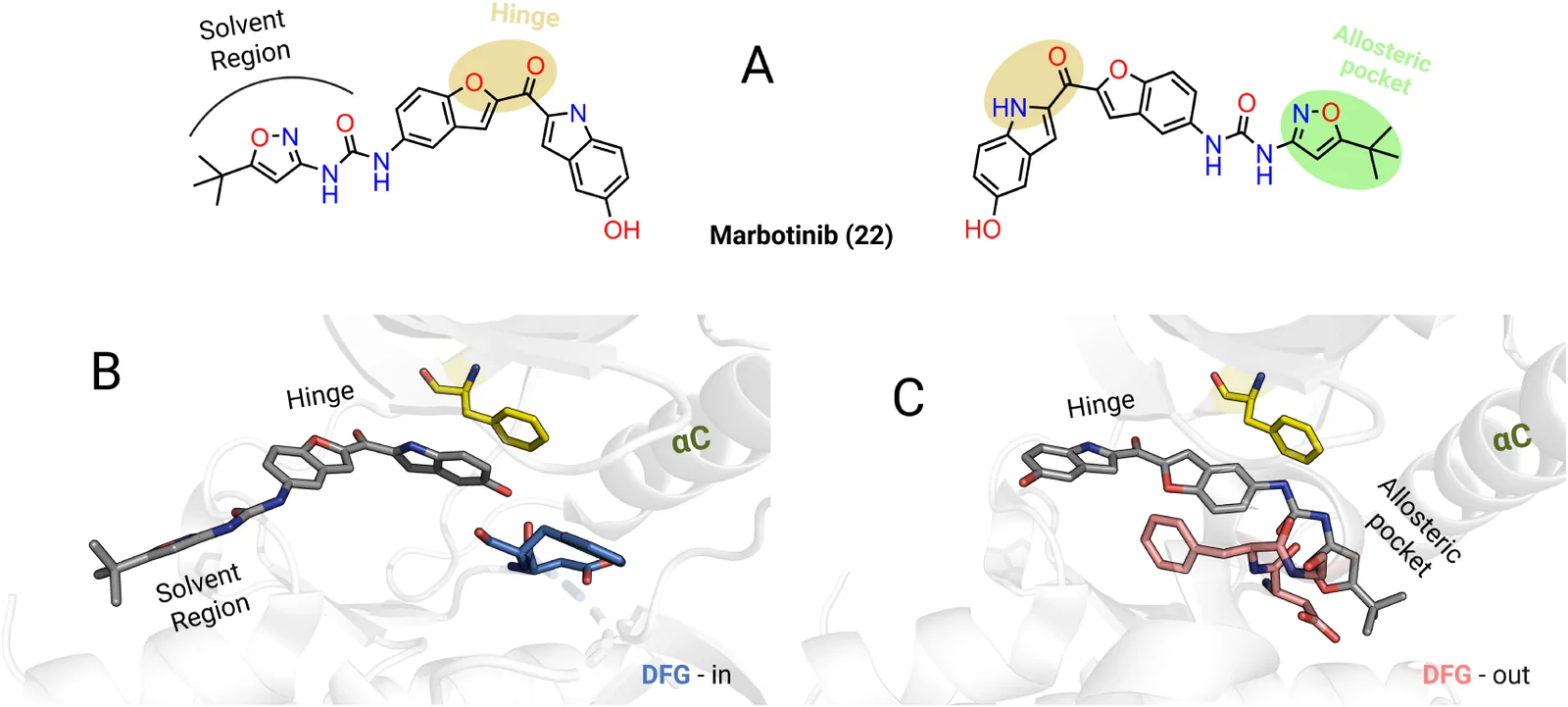
(**A**) Chemical structure of marbotinib (**22**). (**B**) Schematic representation of **22** (PDB: 6JQR) in the active FLT3 conformation (DFG-in). (**C**) Schematic representation of **22** (PDB: 4RT7) in the inactive FLT3 conformation (DFG-out).

Structurally, compound **22** exhibits a longer and more extended molecular scaffold, characteristic of type II FLT3i. At the same time, its architecture allows a different orientation for binding to the DFG-in conformation. In the DFG-out conformation, the *tert*-butyl isoxazole group of **22** extends toward the allosteric cavity. In the DFG-in conformation, the ligand adopts an alternative orientation, approximately rotated by 180° relative to the previous orientation. This unique conformational behavior underlies the classification of **22** as a hybrid FLT3 inhibitor.^107^

Although CHMFL-FLT3-122 (**19**) was initially presented in the context of multitarget inhibitors, as it was developed from the scaffold of the BTK inhibitor ibrutinib (**18**), its discovery paved the way for the development of compounds specifically designed to overcome FLT3-associated resistance mechanisms. From this point onward, subsequent efforts focused not only on optimizing potency against FLT3, but also on achieving activity against clinically relevant secondary mutations. The compound CHMFL-FLT3-213 (**23**; Figure 18) was developed from **19** as an FLT3 inhibitor with activity against secondary mutations, including variants associated with resistance to classical inhibitors. Despite exhibiting promising inhibitory activity against mutant FLT3, with GI_50_ values of <0.3 nM for FLT3-ITD and 2 nM for FLT3-ITD-F691L in BaF3 cells, **23** showed concomitant inhibition of FLT3 and c-KIT, which raised concerns regarding hematological toxicity. As mentioned, FLT3 and c-KIT belong to the same class III receptor tyrosine kinase (RTK) family, implying a high degree of structural similarity, which often leads to overlapping inhibition profile and results in adverse hematological effects, such as myelosuppression. In order to overcome these limitations, the compound **24** (Figure 18) was developed as a structural derivative of **23**, designed to improve selectivity toward FLT3. The introduced structural modifications allowed the preservation of activity against resistant FLT3 mutations, while also promoting improvements in the pharmacokinetic profile, such as increased bioavailability.^6,110–112^

**Figure 18:**
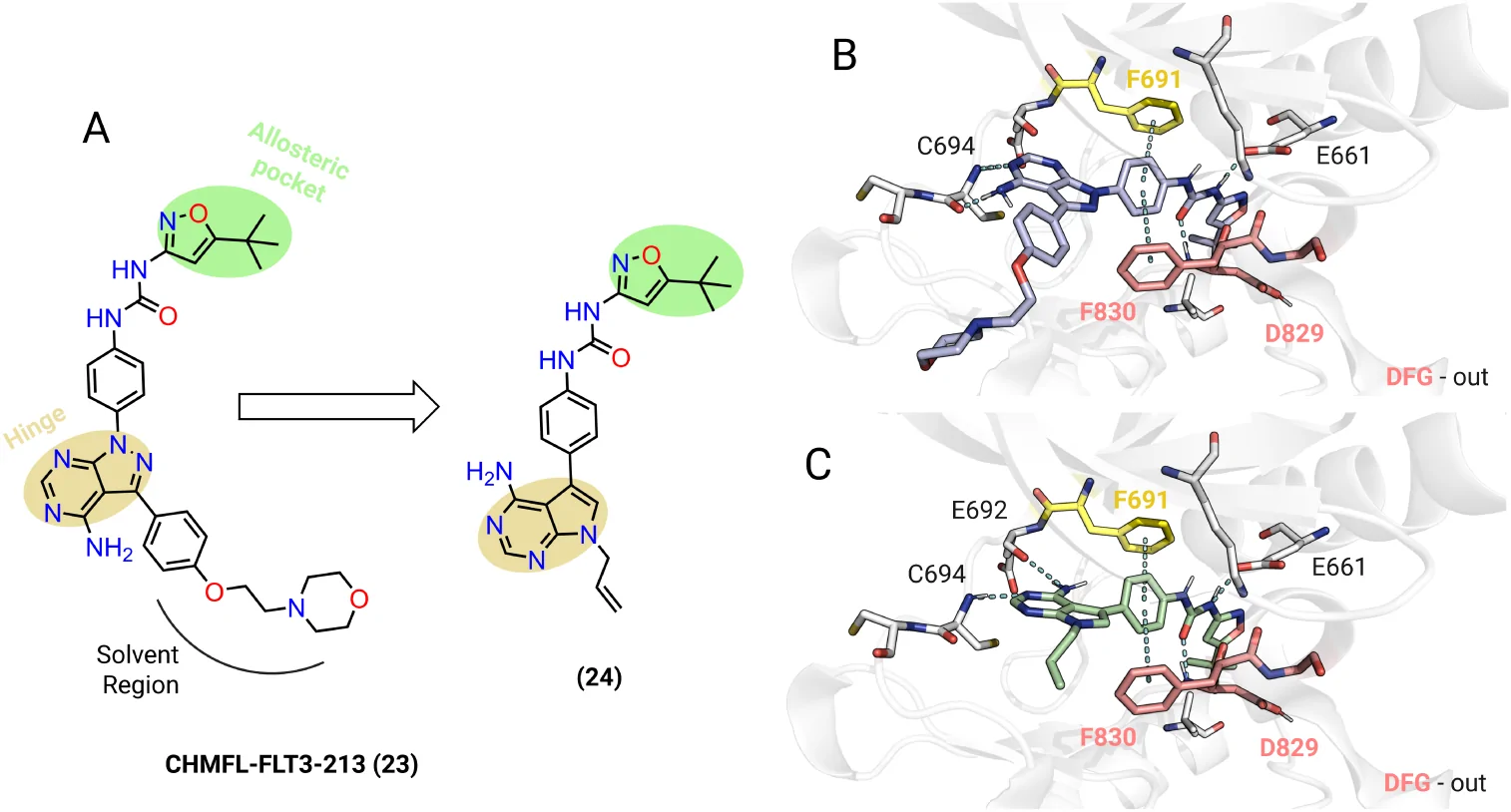
(**A**) Chemical structure and rational design of **24**. (**B**) Docking of CHMFL-FLT3-213 (**23**) (pale violet-colored carbon atoms) into FLT3 kinase (PDB: 4RT7) performed using the GOLD program (CHMPLP: 103.57), under the same conditions described for **15**. (**C**) Docking of **24** (light green-colored carbon atoms) into FLT3 kinase (PDB: 4RT7) performed using the GOLD program (CHMPLP: 103.93) as in **B**.

In cellular assays using the BaF3 cell line, compound **24** exhibited inhibitory activity against both FLT3-ITD (IC_50_: 0.92 nM) and FLT3-ITD-F691L (IC_50_: 12.99 nM), demonstrating efficacy even against resistance-associated variants. Moreover, the compound showed an oral bioavailability of 59.5%, higher than that of its precursor CHMFL-FLT3-213 (**23**) (18.8%). Docking analysis of **24** interactions with FLT3 revealed that the primary amine substituent on its heterocyclic ring forms a hydrogen bond with residue E692 (3.1 Å), while the heterocyclic nitrogen interacts with residue C694 (2.9 Å). In addition, the urea moiety of the ligand engages in hydrogen bonding with residues D829 (2.4 Å) and E661 (2.7 Å). The same interaction pattern is observed for **23**, whose interactions include the involvement of the urea group with D829 (2.5 Å) and E661 (2.6 Å), except for the hydrogen bond with E692, due to the diverse orientation of the primary amine. In **23**, the primary amine interacts with residue C694 (3.0 Å), as does the heterocyclic nitrogen (2.8 Å).^58,59,110^

Protein degradation-based strategies have emerged as a promising approach to overcome resistance mechanisms associated with FLT3 inhibitors, with proteolysis-targeting chimeras (PROTACs) being designed to promote the selective elimination of mutant FLT3 proteins rather than merely suppressing their catalytic activity. Early studies demonstrated the feasibility of this strategy, as quizartinib (**3**) was converted in 2018 into a PROTAC capable of inducing FLT3-ITD degradation in MV4-11 cells (Figure 19A). Subsequently, Chen and colleagues expanded this approach by developing FLT3-targeting PROTACs with activity against multiple mutant forms of the receptor. In addition, other FLT3 inhibitors, such as sunitinib and gilteritinib (**2**), have also been explored as FLT3 ligands in PROTAC-based constructs. Despite these advances, the development of FLT3 degraders with favorable pharmacokinetic properties, particularly oral bioavailability, remains highly challenging. In this context, compound A20 (**25**; Figure 19A), developed through rational design, represents a notable advance. This compound exhibited potent in vivo activity in AML models, efficiently inducing FLT3-ITD degradation with a DC_50_ of 7 nM in MV4-11 cells, while also displaying oral bioavailability of 53%. These findings highlight the emerging therapeutic potential of FLT3-targeted degraders as viable candidates for overcoming acquired resistance.^40,113–115^

**Figure 19:**
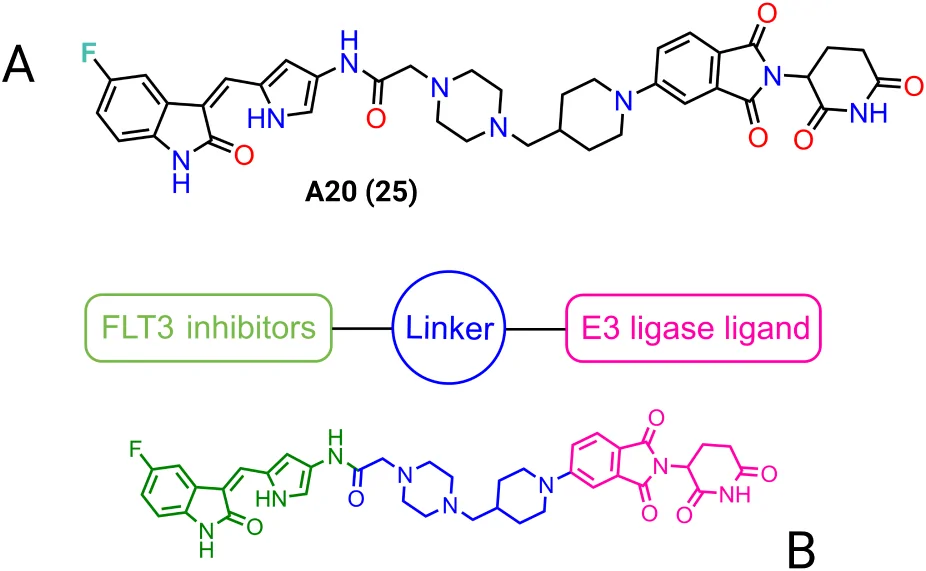
(**A**) Chemical structure of A20 (**25**). (**B**) FLT3-targeting PROTACs: The FLT3 ligand (green) binds to the kinase, while the E3 ligase ligand (pink), connected through a linker (blue), recruits the ubiquitin–ligase complex. This induced proximity promotes FLT3 polyubiquitination, ultimately leading to its proteasomal degradation.

The macrocyclization strategy has also been employed in the development of novel FLT3 inhibitors, emerging as an interesting approach to circumvent resistance mechanisms associated with FLT3 mutations. Macrocyclization enables conformational restriction of the ligands, reducing entropic penalties upon binding and stabilizing bioactive conformations, which may translate into improved pharmacological activity. The macrocyclic inhibitor pacritinib (**12**) represents an example of a macrocycle with inhibitory activity against FLT3, although it exhibits limited efficacy against secondary mutations, such as the gatekeeper F691L mutation. More recently, a pyrazinamide-based macrocyclic FLT3 inhibitor (**27**) was described, demonstrating potent activity against clinically relevant resistance mutations. Compound **27** suppressed the proliferation of BaF3 cells transfected with FLT3-ITD, FLT3-ITD-D835Y, FLT3-ITD-F691L, and FLT3-ITD-D835Y/F691L, as well as the acute myeloid leukemia (AML) cell line MV4-11, with IC_50_ values of 12.2, 10.5, 24.6, 16.9, and 6.8 nM, respectively (Figure 20). **27** was rationally designed based on the binding mode of the prototype compound G-749 (**26**), preserving key interactions with the target. Similar to its precursor, **27** was shown to be capable of forming hydrogen bonds with the hinge region residues C694 (2.9 Å) and E692 (2.6 Å).^58,59,116,117^

**Figure 20:**
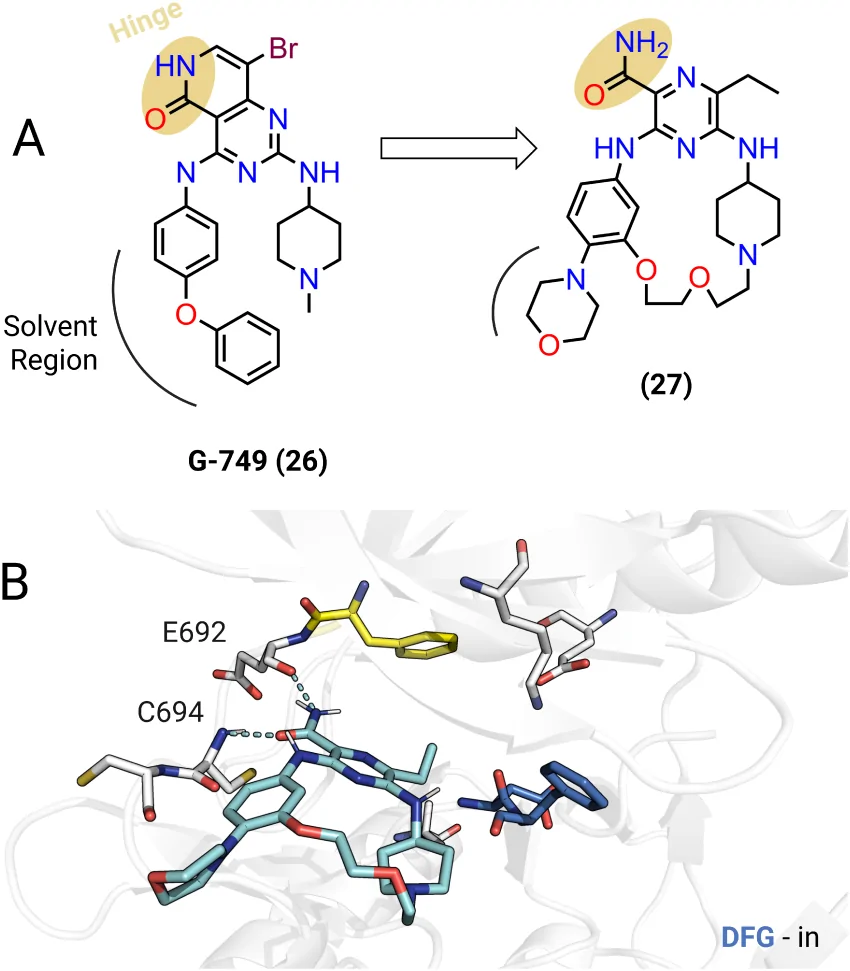
(**A**) Chemical structures of compounds G-746 (**26**, left) and **27** (right). (**B**) Co-crystal structure of **27** (cyan-colored carbon atoms) bound to FLT3 (PDB: 8XB1), with key residues highlighted. Interactions between the ligand and the target are indicated by light blue dashed lines between heavy atoms. Structural visualization and inspection were performed using PyMOL.

## Conclusions

In recent years, several new-generation FLT3 inhibitors have been reported, many of which exhibit improved selectivity and the ability to inhibit resistance-associated mutations, including gatekeeper mutations. These advances represent significant progress in the development of more effective therapies for acute myeloid leukemia; however, important challenges remain. It is well established that FLT3 inhibitors typically occupy the ATP adenine region through nitrogen-containing heterocyclic rings capable of forming hydrogen bonds with residues such as C694 and E692. This structural feature is conserved across different classes of inhibitors discussed herein and represents a key requirement for initial target recognition and inhibitory activity.^6,7,64^

Several type II inhibitors demonstrate strong activity against multiple mutant variants but show reduced efficacy toward the D835Y mutation in the activation loop, which favors the FLT3 active conformation. In contrast, type I inhibitors can bind to the active DFG-in conformation but generally exhibit lower selectivity. Covalent inhibitors offer an innovative and effective approach; however, they require careful monitoring, as mutations in critical binding residues, such as C695, could give rise to resistance mechanisms, as observed with other covalent kinase inhibitors like ibrutinib (**18**) (C481S mutation in BTK).^6,105,118^

To improve the therapeutic landscape, emerging strategies, including PROTACs designed to promote FLT3 degradation and the development of macrocyclic inhibitors, show promise as potential alternatives to overcome resistance to FLT3 inhibitors.^40,113–116^

## Abbreviations

ALK: anaplastic lymphoma kinase
AML: acute myeloid leukemia
ATP: adenosine triphosphate
BCL-2: B-cell lymphoma protein 2
BTK: Bruton’s tyrosine kinase
c-KIT: KIT proto-oncogene receptor tyrosine kinase
CSF1R: colony stimulating factor 1 receptor
DFG: aspartate (D), phenylalanine (F), and glycine (G)
DNA: deoxyribonucleic acid
EGFR: epidermal growth factor receptor
FDA: Food and Drug Administration
FGF2: fibroblast growth factor 2
FLT3: FMS-like tyrosine kinase 3
FL: FLT3 ligand
FLT3i: FLT3 inhibitors
FOXO1: forkhead box protein O1
FOXO3: forkhead box protein O3
HDAC: histone deacetylase
HDACi: HDAC inhibitors
HMAs: hypomethylating agents
ITD: internal tandem duplications
JAK2: Janus kinase 2
JAK3: Janus kinase 3
LTK: leukocyte receptor tyrosine kinase
MAPK: mitogen-activated protein kinase
MCL-1: myeloid cell leukemia-1
PDB: Protein Data Bank; PDGFR-α, platelet-derived growth factor receptor alpha
PDGFR-α: platelet-derived growth factor receptor alpha
PI3K: phosphatidylinositol-3-kinase
PROTAC: proteolysis-targeting chimera
RMSD: root-mean-square deviation
RTK: receptor tyrosine kinase
STAT5A: signal transducer and activator of transcription 5A
TKD: tyrosine kinase domain
VEGFR: vascular endothelial growth factor receptor
